# Machine learning models for predicting vasospasm following ruptured intracranial aneurysms: a systematic review and meta-analysis

**DOI:** 10.1007/s00701-025-06725-y

**Published:** 2025-12-03

**Authors:** Matteo Palermo, Sonia D’Arrigo, Alessandro Olivi, Carmelo Lucio Sturiale

**Affiliations:** 1 Department of Neurosurgery, Fondazione Policlinico Universitario A. Gemelli IRCCS, Università Cattolica del Sacro Cuore, Rome, Italy; 2 Department of Anaesthesia and Intensive Care, Fondazione Policlinico Universitario A. Gemelli IRCCS, Università Cattolica del Sacro Cuore, Rome, Italy

**Keywords:** Vasospasm, Machine learning, Predictive modeling, Artificial intelligence, Radiomics, Diagnostic accuracy, Deep learning

## Abstract

**Background:**

Cerebral vasospasm remains a leading cause of delayed cerebral ischemia following aneurysmal subarachnoid hemorrhage (aSAH). Despite advances in critical care, current monitoring strategies are reactive and non-personalized. Machine learning (ML) has emerged as a promising tool to anticipate vasospasm risk.

**Methods:**

A systematic review and meta-analysis were performed following PRISMA 2020 guidelines. PubMed and Embase databases were searched for studies applying ML algorithms to predict clinical or radiological vasospasm. Data were pooled using bivariate and proportional meta-analyses and their quality was assessed with the PROBAST tool.

**Results:**

Twelve studies (2011–2025) encompassing 25 ML models were included. Deep learning achieved the highest sensitivity (mean: 97.6%) and AUC-ROC (0.97), outperforming regression, ensemble, and SVM methods in sensitivity (p = 0.003) but not in specificity or AUC. SVM models showed the highest NPV (85%), while ensemble and regression methods had superior PPV. Across cohort types, deep learning consistently delivered high accuracy and generalizability, although with greater PPV variability. Bivariate analysis confirmed that artificial neural networks and random forest models achieved favorable sensitivity–specificity trade-offs. Risk of bias was low to moderate, with most concerns related to patient selection and lack of external validation.

**Conclusion:**

ML models, particularly deep learning and ensemble methods, demonstrate promising accuracy in predicting vasospasm after aSAH. These tools may enable earlier, personalized interventions; however, methodological heterogeneity, limited external validation, and lack of prospective trials currently hinder clinical adoption.

**Supplementary Information:**

The online version contains supplementary material available at 10.1007/s00701-025-06725-y.

## Introduction

Cerebral vasospasm following aneurysmal subarachnoid hemorrhage (aSAH) is a feared complication that substantially worsens patient outcomes. Angiographically, vasospasm occurs in a majority of SAH cases, up to 70% of patients, and manifests clinically in roughly 20–40% of patients [6, 11, 15]. It is a primary driver of delayed cerebral ischemia (DCI) and is consistently associated with increased morbidity and mortality. Since the definition proposed by Vergouwen in 2010, the field has progressively distinguished vasospasm from DCI [20]. While vasospasm describes the narrowing of large brain arteries seen on angiography or Doppler, DCI refers to the actual clinical problem, new neurological deficits or a stroke that cannot be explained by other causes. Vasospasm is only one of several processes that can lead to DCI, together with microcirculatory dysfunction, cortical spreading depolarizations, and impaired autoregulation [20].

Indeed, vasospasm after SAH has been linked to poorer outcomes, but these factors alone, such as admission clinical grade or hemorrhage severity scales, have only modest predictive power. Consequently, standard care has focused on intensive monitoring of all SAH patients [1, 5, 12, 19]. Typical management includes prolonged neuro-ICU admission with daily transcranial Doppler ultrasound and periodic CT angiography or perfusion imaging, along with prophylactic oral nimodipine for all patients. These measures detect vasospasm only after it begins, rather than predicting it in advance, and are resource-intensive [5, 15, 16, 18]. In practice, this one-size-fits-all approach means many low-risk patients undergo intensive monitoring, while vasospasm can nonetheless develop unpredictably in others [17].

Machine learning (ML) has emerged as a promising tool to improve early risk stratification in this setting. By integrating large numbers of clinical, physiologic, and imaging variables, ML models may capture complex patterns that elude traditional statistical methods. While several reviews have assessed the use of ML to predict DCI and overall patient outcomes after aSAH, no systematic review to date has focused specifically on ML-based prediction of cerebral vasospasm [9, 14, 25]. The existing literature on vasospasm prediction remains fragmented, with only a handful of ML models reported, most from single centers with modest sample sizes [7, 24]. Given the heterogeneity of algorithms, data sources, and performance metrics, a systematic review and meta-analysis is warranted to synthesize current evidence, compare model performance, and identify methodological gaps in the prediction of vasospasm.

## Methods

**Fig. 1:**
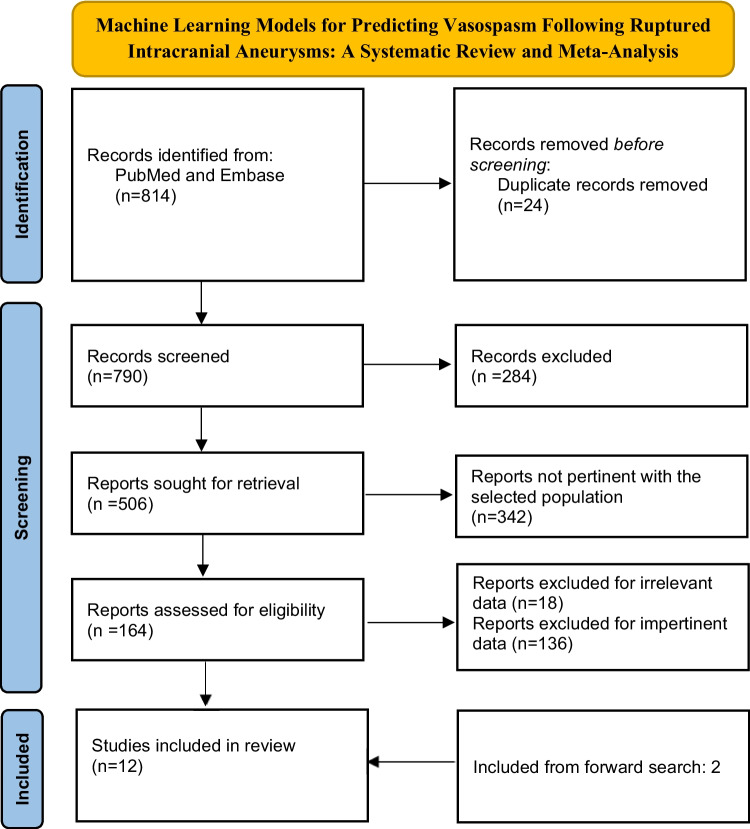
PRISMA 2020 flow diagram of study selection process

This systematic review was conducted in accordance with the PRISMA 2020 (Preferred Reporting Items for Systematic Reviews and Meta-Analyses) guidelines [10]. The research question was structured using the PICO framework: Population – patients with ruptured aneurysms; Intervention – application of machine learning models; Comparison – patients assessed without ML-based predictive tools; Outcome – development of vasospasm (Fig. 1).

### Search strategy

A systematic literature search was conducted in accordance with the Preferred Reporting Items for Systematic Reviews and Meta-Analyses (PRISMA) guidelines. The search was performed in the PubMed/MEDLINE and Embase databases on July 18th, 2025, using the following search strategy: (machine learning OR artificial intelligence OR deep learning OR neural network OR random forest OR support vector machine OR supervised learning OR unsupervised learning) AND (aneurysm* OR vasospasm* OR SAH OR subarachnoid) AND (brain OR intracranial OR cerebral).

### Selection criteria

Studies were included if they met the following criteria: (1) original research articles; (2) investigated the use of machine learning (ML) models to predict radiological or clinical vasospasm; (3) utilized real-world clinical data; (4) employed imaging-based and/or clinical-based features (age, sex, comorbidities) as model inputs; and (5) reported at least one model performance metric, such as sensitivity, specificity, area under the receiver operating characteristic curve (AUC-ROC), positive predictive value (PPV), or accuracy.

Exclusion criteria were as follows: (1) review articles, editorials, case reports, or conference abstracts; (2) studies using non-clinical or non-imaging-based input features; (3) non-English language publications; and (4) studies based on simulated or synthetic data.

### Study selection and data extraction

Screening of the titles and abstracts of retrieved articles was performed by 2 independent reviewers (MP, CLS). Full-text articles of potentially relevant studies were then reviewed for final inclusion. Any discrepancies between reviewers were resolved through discussion or involvement of a third reviewer for consensus (AO). The reference lists of the included studies were manually searched to identify additional records. Data extraction was performed using a standardized Excel workbook, including author, year of publication, number of patients included in the training and validation cohorts, mean age of patients in the validation group, machine learning algorithms employed, predictive features used for modeling, and the reported model performance metrics. In cases where one study reported data on several ML algorithms, separate entries were considered for each of the algorithms. Conversely, if the study demonstrated progressive improvement of performance through the addition of parameters or features, we only included the final and best-performing version of the algorithm. Performance matrices were used to back-calculate the confusion matrices. In this review, we included only studies that applied ML specifically to predict cerebral vasospasm following aSAH, rather than DCI or general clinical outcomes. Although vasospasm is a well-known contributor to DCI, it is not synonymous with it. Therefore, studies that focused solely on DCI were included only if they also provided specific results or predictive modeling for vasospasm.

### Quality assessment

Risk of bias was assessed for individual studies using an adapted version of the PROBAST (Prediction model Risk Of Bias ASsessment Tool) [21]. PROBAST was developed to determine bias in published prediction models.. This tool assesses each study across four domains: partecipants, predictors, outcome and analysis (Table 1 – Suppl. Material). Applicability domain cannot be assessed in systematic reviews.. Two independent reviewers (MP and CLS) conducted the assessment. Any discrepancies were resolved through discussion with a third reviewer (AO).

### Statistical analysis

All statistical analyses were performed to compare the performance of different ML algorithms across key diagnostic metrics, including sensitivity, specificity, accuracy, positive predictive value (PPV), negative predictive value (NPV), diagnostic odds ratio (DOR), and area under the receiver operating characteristic curve (AUC-ROC). Continuous outcome variables were first tested for normality. For normally distributed metrics, one-way analysis of variance (ANOVA) was conducted to assess differences across algorithms. In cases where the assumption of normality was not met or where sample sizes were small or unbalanced, the Kruskal–Wallis test was applied.

For statistically significant ANOVA results (*p* < 0.05), Tukey's Honest Significant Difference (HSD) test was used for post hoc pairwise comparisons. Similarly, for significant Kruskal–Wallis tests, pairwise comparisons were performed using Dunn’s test, with p-values adjusted for multiple comparisons using the Holm-Bonferroni correction.

To evaluate model generalizability, descriptive statistics were also computed separately for the training, test, and validation cohorts. These included means, standard deviations, and coefficients of variation for each diagnostic metric stratified by algorithm and cohort. All analyses were conducted using JASP version 19.3.0, and *p*-values less than 0.05 (after adjustment) were considered statistically significant.

Subsequently, we conducted a bivariate analysis of sensitivity versus 1–specificity to generate summary performance estimates across all included studies. This analysis was performed without stratifying by cohort type (i.e., training, test, or validation), in order to represent the performance of individual algorithms.

## Results

The search yielded 814 articles in total. During the initial screening, we excluded non-English publications (14), duplicates (24), reviews (123), studies that did not involve vasospastic patients (147), those not focused on the target population (342), and articles not relevant to the topic (18). This left 164 studies specifically involving patients with aSAH. After a more detailed review, we excluded another 154 articles due to insufficient or irrelevant data. We also identified 2 additional studies through forward citation searching. In the end, 12 studies met all the inclusion criteria and were included in the final analysis (Fig. 1; Table 1). Their quality was assessed using the PROBAST tool to evaluate risk of bias (Table 2). The full selection process is summarized in the PRISMA 2020 flowchart (Fig. 1), which outlines each stage: identification, screening, eligibility, and final inclusion.

**Table 1:** Machine learning details and performances

Author Year	Models	Train (CV)	Ext- Val	Test	H/CFD	M	DL	R	Train	Test	Val
Dumont et al., 2011 [1]	ANN	91	N/A	22	N	Y	Y	N	**ANN** (SEN = 93.5, SPEC = 91.7, ACC = 92.3, DOR = 159.5, PPV = 85, NPV = 96)	**ANN** (SEN = 100, SPEC = 85.7, ACC = 90.9, AUC = 0.96, PPV = 80, NPV = 100) **LR** (SEN = 85.7, SPEC = 100, ACC = 95.5, AUC = 9.33, PPV = 26, NPV = 93) **LR** (SEN = 50, SPEC = 100, ACC = 81.8, AUC = 0.897, PPV = 100, NPV = 78)	NR
Dumont et al. 2016 [2]	ANN	91	25	22	N	Y	Y	N	**ANN** (SEN = 93.5, SPEC = 91.7, ACC = 92.3, DOR = 159.5, PPV = 85, NPV = 96)	**ANN** (SEN = 100, SPEC = 85.7, ACC = 90.9, AUC = 0.96, PPV = 80, NPV = 100)	**ANN** (SEN = 100, SPEC = 84, ACC = 88, PPV = 67, NPV = 100)
Urbanos et al., 2025 [3]	XGB, RF, ET	403	403	41	Y	Y	N	Y	NR	**XGB + RF + ET** (SEN = 50, SPEC = 61.5, ACC = 63, DOR = 3.48, AUC = 0.596, PPV = 45, NPV = 81) **XGB + RF + ET** (SEN = 66, SPEC = 62, ACC = 58.5, DOR = 1.8, AUC = 0.66, PPV = 39, NPV = 74)	NR
Yang et al., 2025 [4]	RF, LR	122	N/A	53	Y	N	N	Y	NR	**LR**(SEN = 90.5, SPEC = 91.2, ACC = 90.8, DOR = 98.6, AUC = 0.955, PPV = 90, NPV = 91) **RF** (SEN = 91.7, SPEC = 91.2, ACC = 91.4, DOR = 90.9, AUC = 0.966, PPV = 91, NPV = 92)	N/A
KH Kim et al., 2021 [5]	RF	274	N/A	74	N	N	N	Y	N/A	**RF** (SEN = 78, SPEC = 77, ACC = 86, DOR = 23.4, AUC = 0.88, PPV = 53, NPV = 95)	NR
Scherer et al., 2019 [6]	RF	N/A	N/A	125	Y	N	N	Y	N/A	**RF** (SEN = 72.1, SPEC = 66.7, ACC = 71.2, DOR = 5.17, AUC = 0.74, PPV = 91, NPV = 33)	N/A
Street et al., 2023 [7]	LR	N/A	N/A	106	Y	N	N	Y	N/A	**LR** (SEN = 80, SPEC = 50, ACC = 71.4, DOR = 4, AUC = 0.89, PPV = 80, NPV = 50)	N/A
Skoch et al., 2017 [8]	ANN	91	16	22	N	N	Y	Y	**ANN** (SEN = 93.5, SPEC = 91.7, ACC = 92.3, DOR = 159.5, PPV = 85, NPV = 96)	**ANN** (SEN = 100, SPEC = 85.7, ACC = 90.9, AUC = 0.96, PPV = 80, NPV = 100)	**ANN** (SEN = 100, SPEC = 100, ACC = 100, PPV = 100, NPV = 100)
Zarrin et al., 2024 [9]	LightGBM, LR	1750	1654	1750	N	N	Y	N	N/A	**LightGBM** (AUC = 0.88); **LR** (AUC = 0.76)	**LightGBM** (AUC = 0.88)
Sen et al., 2025 [10]	LSTM	N/A	N/A	424	N	N	Y	Y	N/A	**LSTM** (ACC = 76.6, PPV = 68.3)	N/A
Lintas et al., 2024 [11]	SVM	87	N/A	22	N	Y	Y	Y	**SVM** (SEN = 35, SPEC = 88, ACC = 75.3, DOR = 4.06, PPV = 56.2, NPV = 76.1)	**SVM** (SEN = 80, SPEC = 88.2, ACC = 86.4, DOR = 30, PPV = 66.7, NPV = 93.8)	N/A
Roederer et al., 2014 [12]	NB, LR	81	81	NR	Y	Y	Y	Y	**NB**(SEN = 42.9%, SPEC = 73.9%, ACC = 60.5%, DOR = 2.12, AUC = 0.625, PPV = 56%, NPV = 63%) **LR**(SEN = 54.3%, SPEC = 43.5%, ACC = 48.1%, DOR = 0.91, AUC = 0.459, PPV = 42%, NPV = 56%)	NR	NR

*ANN* Artificial Neural Network, *LR* Logistic Regression, *RF* Random Forest, *XGB* Extreme Gradient Boosting, *ET* Extra Trees, *SVM* Support Vector Machine, *NB* Naive Bayes, *LSTM* Long Short-Term Memory, *LightGBM* Light Gradient Boosting Machine, *SEN* Sensitivity, *SPEC* Specificity, *ACC* Accuracy, *DOR* Diagnostic Odds Ratio, *AUC* Area Under the Curve, *PPV* Positive Predictive Value, *NPV* Negative Predictive Value, *H* Hemodynamic parameter, *CFD* Computational Fluid Dynamics-based feature, *M* Morphological parameter, *DL* Deep Learning-based feature, *R* Radiomic parameter, *NR* Not Reported, *Train (CV)* Training set with Cross-Validation, *Ext-Val* External Validation cohort, *Test* Test cohort

**Table 2:** Algorithm families based on their primary characteristics

Family	Algorithms	Description
Regression models	LR, Lasso Regression (Lasso), Ridge Regression, Elastic Net Regression, GLM, GLMNet	Linear models are a class of models that assume a linear relationship between the input variables (features) and the target variable (output). They are widely used for regression tasks, each introducing different forms of regularization or handling of specific types of data.
Instance-based methods	KNN	Instance-based methods make predictions based on similarity measures between instances. They store training examples and classify new data points based on proximity in feature space.
SVM	SVM	SVM is a powerful supervised learning algorithm used for classification and regression tasks. It finds a hyperplane in an N-dimensional space that best separates classes or predicts continuous outcomes, aiming to maximize the margin between classes.
Decision trees	DT	Decision Trees recursively partition data into subsets based on features that best split the data according to certain criteria. They are intuitive and can handle both numerical and categorical data, often used for classification and regression.
Ensemble methods	RF, AdaBoost, XGBoost, LightGBM, GBM, Bagging (e.g., Bagged Decision Trees), BART, CatBoost	Ensemble methods combine predictions from multiple models to improve overall performance. They utilize combinations of decision trees or other base models to achieve better predictive accuracy and robustness.
Generative models	Naive Bayes, LDA, GANs, Variational Autoencoders (VAEs), HMMs	Generative models aim to learn the underlying distribution of data to generate new samples. Naive Bayes and LDA are often used for classification and topic modeling, while GANs and VAEs are powerful tools in image synthesis, data augmentation, and anomaly detection. HMMs are widely used in sequence modeling.
Deep learning	CNN, ANN, ResNet, MLP, transformers (e.g., BERT, GPT)	Deep learning involves neural networks with multiple layers that automatically learn hierarchical representations of data. CNNs are particularly effective for image processing, ANNs for general tasks, ResNet for deep architectures, MLP for multi-layer perceptrons, and Transformers for sequential data processing.

*ANN* Artificial Neural Network, *BART* Bayesian Additive Regression Trees, *CNN* Convolutional Neural Network, *DT* Decision Trees, *GBM* Gradient Boosting Machine, *GLM* Generalized Linear Models, *KNN* K-Nearest Neighbors, *LR* Linear Regression, *MLP* Multilayer Perceptron, *RF* Random Forest, *SVM* Support Vector Machine

### Systematic review and meta-analysis

We included 12 studies published between 2011 and 2025 (Table 1) in our systematic review. Most originated from Asia or North America, with algorithms developed exclusively in retrospective datasets. A total of 25 machine learning models from distinct studies were analyzed, encompassing various families such as SVM Models, Generative Models, Regression Models, Ensemble Methods and Deep Learning (Table 2).

### Stratification by algorithm

Deep learning models achieved the highest mean accuracy across cohorts (90.6% ± 5.6%), followed by SVM (80.9% ± 7.8%), regression methods (64.4% ± 32.7%), and ensemble methods (51.2% ± 41.3%). A two-way ANOVA revealed no statistically significant difference in accuracy between algorithm groups (*p* = 0.070) (Table 3, Table 2—Suppl. Material). In particular, the accuracy of deep learning models did not significantly differ from that of ensemble methods (MD = 39.4%, *p* = 0.082) or regression methods (MD = 26.2%, *p* = 0.446). All other pairwise comparisons were non-significant as well (adjusted *p* > 0.05).

**Table 3:** Aggregated diagnostic metrics (Sensitivity, Specificity, AUC-ROC, PPV, NPV) stratified by algorithm type

Algorithm	N	Mean	SD	SE	Coefficient of variation
Descriptives—sensitivity					
Deep Learning	8	97.563	3.364	1.189	0.034
Ensemble Methods	4	76.950	10.986	5.493	0.143
Regression Methods	6	68.717	19.303	7.881	0.281
SVM Model	2	57.500	31.820	22.500	0.553
Descriptives—specificity					
Deep Learning	8	89.525	5.323	1.882	0.059
Ensemble Methods	4	74.225	12.935	6.467	0.174
Regression Methods	6	74.367	25.728	10.503	0.346
SVM Model	2	88.100	0.141	0.100	0.002
Descriptives—AUC-ROC					
Deep Learning	3	0.973	0.023	0.013	0.024
Ensemble Methods	6	15.354	35.589	14.529	2.318
Regression Methods	5	0.732	0.205	0.092	0.280
Descriptives—PPV					
Deep Learning	9	53.974	40.191	13.397	0.745
Ensemble Methods	4	0.685	0.266	0.133	0.388
Regression Methods	6	0.638	0.301	0.123	0.471
SVM Model	2	61.450	7.425	5.250	0.121
Descriptives—NPV					
Deep Learning	8	61.375	50.023	17.686	0.815
Ensemble Methods	4	0.735	0.285	0.143	0.388
Regression Methods	6	0.748	0.179	0.073	0.240
SVM Model	2	84.950	12.516	8.850	0.147

Deep learning models exhibited near-perfect sensitivity, with a mean of 97.6% (SD 3.4%). In contrast, ensemble methods and regression-based models showed lower sensitivities around 77.0% ± 11.0% and 68.7% ± 19.3%, respectively. SVM had the lowest sensitivity (57.5% ± 31.8%) among the groups. These differences were statistically significant overall (*p* = 0.003). Post hoc tests confirmed that deep learning achieved significantly higher sensitivity than both regression methods (MD = 28%, *p* = 0.011) and SVM (MD = 40%, *p* = 0.017). The sensitivity of ensemble methods was intermediate; it was not significantly different from deep learning (*p* = 0.196), nor from regression methods or SVM (all *p* > 0.8).

Specificity was high for both deep learning and SVM models, with mean values of 89.5% ± 5.3% and 88.1% ± 0.1%, respectively. Ensemble and regression methods had lower specificities on average (74.2% ± 12.9% and 74.4% ± 25.7%) and showed greater variability. However, an ANOVA found no significant effect of algorithm type on specificity (*p* = 0.256). None of the pairwise differences in specificity between algorithm groups reached statistical significance after correction (all *p* > 0.5).

Deep learning attained the highest area under the ROC curve, with a mean AUC-ROC of approximately 0.973 ± 0.023. Regression-based models achieved a moderate AUC (0.732 ± 0.205). In contrast, the ensemble methods group showed an anomalously low average AUC (0.15, SD 0.36), indicating that some ensemble models performed poorly in terms of ranking positives vs. negatives. Statistically, these AUC differences were not significant (*p* = 0.551), likely due to the high variance and small N in the ensemble category. Consistent with the ANOVA, post hoc comparisons found no significant pairwise differences in AUC-ROC between the algorithm groups (all adjusted *p* = 1.00).

Positive predictive value (precision) varied significantly by algorithm (*p* = 0.003). Deep learning models had the lowest mean PPV (54.0% ± 40.2%), indicating a relatively high false-positive rate, whereas ensemble methods and regression methods achieved higher mean PPVs (68.5% ± 26.6% and 63.8% ± 30.1%, respectively). SVM models also showed high precision (61.5% ± 7.4%) despite the small sample. Post hoc tests revealed that deep learning’s PPV was significantly lower than that of both ensemble methods (*p* = 0.031) and regression methods (*p* = 0.012). In contrast, there was no significant difference in PPV between ensemble and regression models (*p* = 1.000). SVM’s precision did not differ significantly from the other groups (all *p* > 0.1).

A significant effect of algorithm type was also found for negative predictive value (*p* = 0.004). Deep learning models yielded the lowest NPV (61.4% ± 50.0%), reflecting considerable variability in their ability to rule out negative cases. Ensemble and regression methods showed higher and more consistent NPVs, approximately 73–75% with SD 18–29%. The highest NPV was achieved by SVM models (85.0% ± 12.5%). In post hoc comparisons, SVM models had a significantly higher NPV than regression-based models (*p* = 0.041). Deep learning’s NPV was significantly lower than that of regression methods as well (*p* = 0.023). The difference between deep learning and ensemble methods approached significance (deep lower, *p* = 0.053), whereas the ensemble vs. regression NPV was virtually identical (*p* = 1.000). No other pairwise differences reached significance.

### Stratification by cohort type and algorithm

Across all metrics, deep learning models demonstrated strong performance on both test and validation cohorts, often achieving the highest means, especially for sensitivity, accuracy, and AUC-ROC, with low variability, although their precision (PPV) and test NPV showed more inconsistency. Ensemble and regression methods tended to exhibit higher variability in performance with high SD/CV in accuracy, AUC, PPV. Single-model categories (SVM and generative models) yielded outcomes that, while lacking variability data, highlighted some extremes: for instance, the generative approach often underperformed (42.9% sensitivity in training), and the SVM excelled in certain aspects (93.8% NPV in test) (Table 4, Table 3 – Suppl. Material).

**Table 4:**
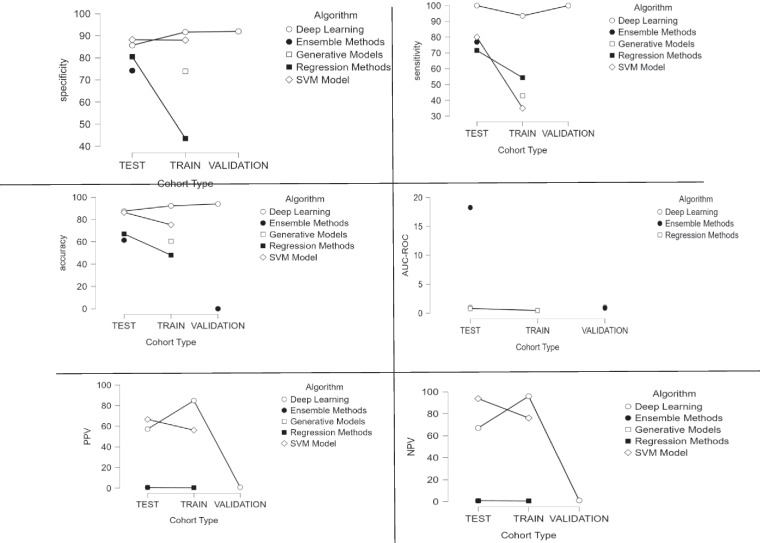
Stratification of performance metrices by algorithm and cohort type

### Bivariate analysis

**Fig. 2:**
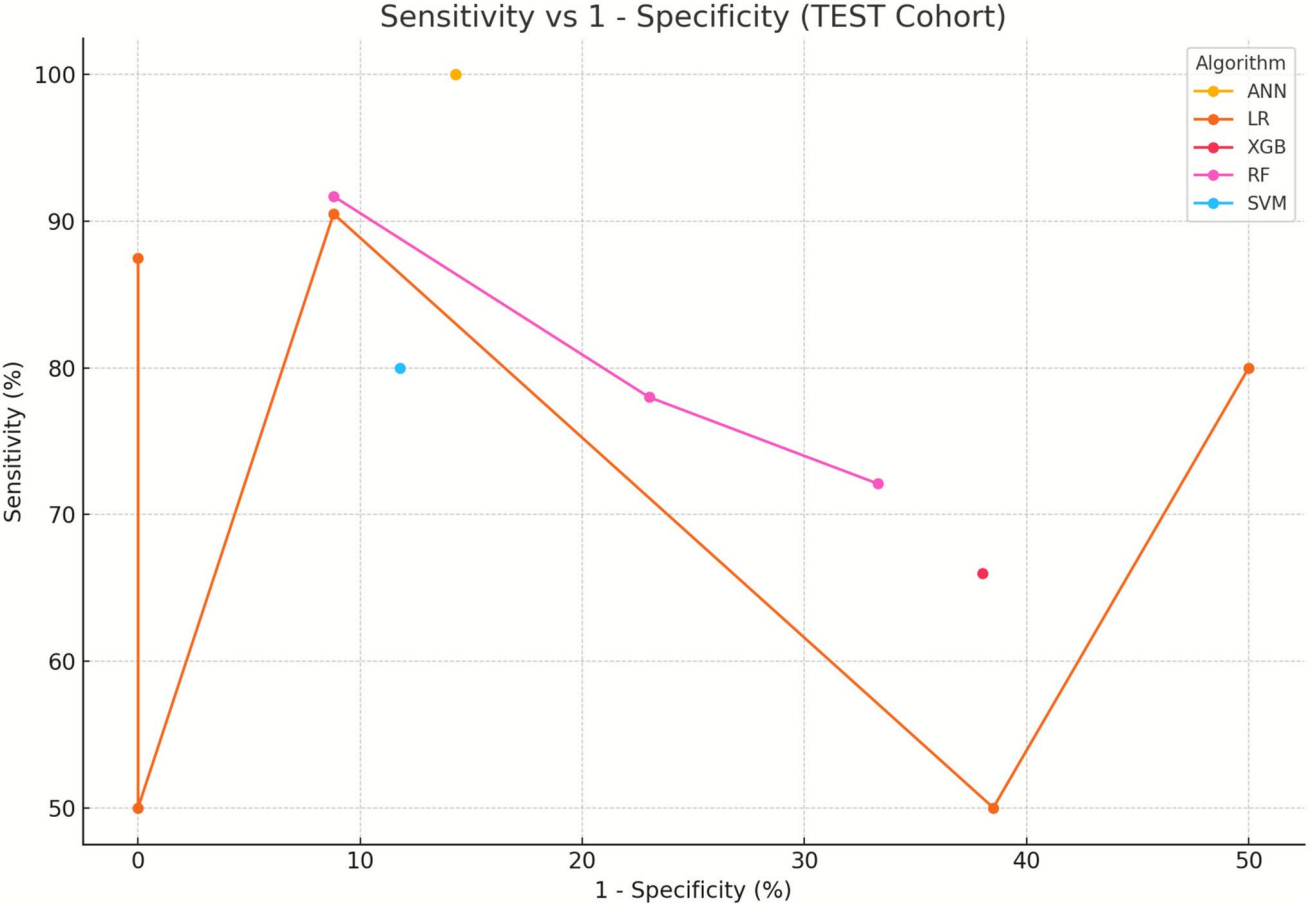
Bivariate analysis of sensitivity and false positive rate (1-specificity) across machine learning algorithm types (TEST cohort)

Then, we performed a bivariate analysis of the average sensitivity and false positive rate (1-specificity) across the main algorithmic types (Fig. 2). Artificial Neural Networks (ANN) achieved the highest sensitivity (100%) with moderate false positive rates (1—specificity = 15%), logistic regression (LR) models exhibited the widest dispersion across the plot. While one LR model achieved near-perfect specificity (1—specificity = 0%) at the cost of low sensitivity (50%), other LR models shifted toward more balanced trade-offs. Random forest (RF) models clustered in the upper-central region of the plot, showing consistently high sensitivity (72–92%) but with increased false positive rates (1—specificity = 22–33%), XGBoost (XGB) models achieved moderate sensitivity (66%) but showed relatively high 1—specificity (38%), indicating limited discriminative ability in this cohort. Support Vector Machines (SVM) reached a good balance with sensitivity of 80% and 1—specificity around 12%, reflecting strong discriminatory performance with a low false positive burden.

### Risk of bias

Risk of bias in the results synthesis was generally low across studies. Most studies (8 out of 12) demonstrated low risk of bias in patient selection due to well-defined inclusion criteria and representative samples. However, three studies [2, 3, 16] were rated as high risk in this domain due to retrospective design or selective sampling. The index test domain showed unclear risk in four studies, primarily due to insufficient reporting regarding blinding or test threshold pre-specification, while all studies were consistently rated as low risk for both the reference standard and the flow and timing domains..

## Discussion

Cerebral vasospasm is a common and serious complication after aSAH, occurring in roughly 20–30% of adult patients and contributing significantly to delayed cerebral ischemia [11]. Across the twelve studies reviewed, our meta-analysis suggests that ML models can achieve moderate to high discrimination in predicting vasospasm risk, though performance varies by cohort size and design. Notably, five studies (5/12) incorporated hemodynamic or computational fluid dynamic features, four (4/12) used vascular morphology as a predictor, seven (7/12) applied deep learning–based approaches, and nine (9/12) included radiomic parameters in their models. Dumont et al. (2011) demonstrated that an ANN could predict symptomatic vasospasm in adults with excellent accuracy, yielding an area under the ROC curve (AUC) of 0.96, significantly surpassing traditional logistic regression models (AUC = 0.90) [3]. Similarly, a more recent multi-center study by Zarrin et al. (2024) used a gradient-boosting algorithm (LightGBM) trained on 172 intensive-care variables to predict clinically significant vasospasm requiring intra-arterial verapamil [24]. This model achieved high performance, AUC = 0.88 at one-week advance prediction, and generalized well across two institutions. Naïve Bayes and logistic classifiers have also been used [24]. For example, Roederer et al. (2014) trained a Naïve Bayes model on passively collected ICU data (e.g. blood pressure, heart rate, intracranial pressure) and found that it modestly predicted angiographic vasospasm (AUC = 0.71), outperforming models based on standard neurological exams, transcranial Doppler, or perfusion imaging (AUC = 0.50) [13]. In validation studies, ML models often showed high sensitivity but sometimes lower positive predictive value. Dumont’s later work (2016) prospectively applied an ANN at a second center: it achieved 100% sensitivity and 84% specificity for symptomatic vasospasm, indicating perfect negative predictive value but some false positives [2]. Overall, these findings suggest that ML methods can effectively integrate complex clinical data to identify vasospasm risk, often exceeding the performance of traditional regression models. Instead, the largest studies tend to report more moderate AUCs (0.85–0.90) than smaller proof-of-concept studies, reflecting more realistic performance [24].

### Imaging and radiomics predictors

Quantitative neuroimaging features have emerged as powerful predictors of vasospasm risk. A common theme is that the volume and distribution of blood on admission CT scans are strongly prognostic. Street et al. (2023) used a semi-automated segmentation pipeline (ITK-SNAP) to measure initial subarachnoid blood volume and found that each additional 1 cm^3^ of blood increased the odds of later radiographic vasospasm by 7% (OR = 1.069), and that total blood volume predicted vasospasm with AUC = 0.86, significantly outperforming the modified Fisher grade (AUC = 0.70) [17]. Importantly, the measurements were taken from the very first CT scan obtained at presentation, so the quantified blood volume directly reflected the initial hemorrhagic burden. Patients with larger blood volumes not only had a higher risk of developing vasospasm, but also tended to experience more severe changes on Doppler (higher Lindegaard ratios), longer-lasting vasospasm episodes, a greater number of separate episodes, and a longer overall hospital stay. Scherer et al. (2020) similarly performed volumetric analysis, showing that larger SAH clot volumes were associated with higher vasospasm risk (OR = 1.06 per mL) while greater cerebrospinal fluid (CSF) volume was protective (OR = 0.99 per mL) [15]. In addition to volumetric measures, their models also integrated clinical variables such as hemodynamic and radiomic features. These CT-based measures outperform simple qualitative scales because they provide an objective assessment of hemorrhage burden. Beyond volume, radiomics approaches extract detailed texture and shape features. In the largest study reviewed, Urbanos et al. (2025) applied radiomic analysis to segmentations of brain parenchyma and hemorrhage in 403 patients [18]. Their ML models achieved AUCs of 0.75 for predicting vasospasm on held-out data, comparable to clinical-data models [18]. Radiomic features related to heterogeneity and intensity were among the top contributors according to SHAP analysis. Notably, radiomics combined with clinical variables generally predicted outcomes better than vasospasm itself, suggesting that vasospasm remains harder to predict [15, 17, 18].

### Clinical grading and biomarkers

Traditional clinical scores remain important covariates in predictive models. Many studies included measures such as Hunt–Hess grade or WFNS, modified Fisher grade, Glasgow Coma Scale (GCS), and angiographic findings. Kim et al. (2021) used an explainable random-forest model on 343 aSAH patients, and SHAP analysis revealed that aneurysm size was the single strongest risk factor for angiographic vasospasm, contributing 27.6% to the model, followed by patient age (20.7%) and admission GCS [4]. Modified Fisher grade and GCS had relatively smaller contributions. Yang et al. (2025) performed a biomarker study in 175 patients and identified GCS alongside several serum markers, including hypoxia-inducible factor 1α, vascular endothelial growth factor (VEGF), and endothelin-1, as independent predictors of vasospasm [23]. Their multivariable logistic and random-forest models achieved very high AUCs (0.96), highlighting the potential of biochemical markers. Other analyses noted that intraventricular hemorrhage and inflammation-related markers are also associated with vasospasm. In most models, higher clinical severity, lower GCS or higher Hunt–Hess, and poorer neurological grade portended higher vasospasm risk. Taken together, these findings reinforce that both patient-level factors and acute clinical status are key inputs to any predictive algorithm. Some studies also examined treatment or medication effects: for example, in the XAI model by Kim et al., use of postoperative antiplatelet agents (aspirin, cilostazol) was associated with a somewhat lower probability of vasospasm, although this effect was not the main focus [4].

### Pediatric versus adult populations

Cerebral vasospasm is much less common in pediatric aSAH, and only one study in this set focused on children [8]. Skoch et al. (2017) applied an ANN, trained on adult data, to 16 pediatric patients and found it could perfectly separate the single child who developed symptomatic vasospasm from the 15 who did not [16]. This suggests that adult-derived models may have some utility even in younger patients. However, the pediatric study’s sample was very small and the authors note that vasospasm in children is rare but serious, with no established monitoring guidelines. Thus, while machine learning shows promise in children, larger multi-center pediatric datasets would be needed to confirm these results. In general, the adult studies comprised the bulk of the evidence; differences in physiology, comorbidities, and aneurysm etiology between children and adults mean that dedicated pediatric research is a key future direction.

### Limitations

This review highlights several key limitations affecting the current evidence base. First, there was marked variability in performance metrics across algorithm families, with ensemble and regression methods showing high standard deviations that hinder interpretability. Despite their strong sensitivity, deep learning models demonstrated inconsistent precision and NPV, raising concerns about clinical false alarms or missed detections [17, 22, 24]. Most of the reported ML models are retrospective and based on limited samples [3, 16].

Moreover, ML methods that use raw electronic health record data offer practical advantages. Unlike prior models that depend on manually scored imaging scales or radiographic interpretations, modern ML pipelines can consume routinely collected ICU data automatically [24]. This removes subjectivity and enables real-time risk updates as new data arrive. In the Zarrin study, for example, the ML tool continuously stratified patients’ vasospasm risk each day of ICU stay, which could theoretically guide adjustments in monitoring intensity [24]. Finally, the best-performing models have demonstrated robustness across diverse settings: Zarrin et al*.* noted that their model maintained high accuracy at two separate centers with different patient demographics and treatment practices, suggesting good generalizability [24].

Data partitioning strategies were often poorly reported, making it difficult to assess generalizability across training, test, and validation cohorts. In some studies, the assessed metrics showed equal or even better performance on validation than training datasets (Table 4), a counterintuitive result likely due to very small validation cohort, raising concerns about the statistical power of these studies. Some ensemble models exhibited anomalously poor AUC-ROC values, suggesting unstable discriminatory power. Additionally, most studies did not incorporate interpretable machine learning methods, reducing clinical transparency and adoption potential [24]. Class imbalance due to low vasospasm incidence further limited model robustness. Pediatric data were extremely limited, with only one small study available, precluding any firm conclusions in this population. Only a few efforts have included external validation and calibration and none has been tested prospectively [2, 13, 24]. Nonethless, both clinically and angiographically detected vasospasms were included, which may contribute to heterogeneity and requires cautious interpretation of the findings. Finally, PPV and NPV (Table 4) appeared to collapse towards zero in the validation cohorts, most likely due to the very small sample sizes. These results should therefore be interpreted with caution and emphasize the need for larger, prospectively validated cohorts to provide more stable and generalizable estimates of predictive performance.

Regarding clinical applicability, ML tools hold promise but face barriers. In principle, accurate risk stratification could allow intensivists to tailor monitoring: high-risk patients could receive more aggressive surveillance while low-risk patients might have fewer invasive tests [24]. This could improve care efficiency. Nonetheless, integration into practice is nontrivial. Regulatory and implementation challenges remain, including the need for prospective clinical trials and user-friendly decision support interfaces. As one group cautioned, ML outputs “must be interpreted by clinicians” because blind acceptance of AI predictions can lead to errors and liability [24]. Physician trust, explainability, and workflow adaptation are ongoing challenges. Moreover, most published models are not yet packaged for bedside use, and healthcare systems vary in data infrastructure. Thus, despite strong retrospective results, real-world adoption will require rigorous validation and demonstration of impact on patient outcomes.

## Conclusion

Recent studies show that data-driven models, especially those using imaging, clinical, and biomarker inputs, can effectively predict cerebral vasospasm after aSAH. While machine learning often outperforms traditional methods, clinical translation will require prospective validation and standardized risk stratification tools.

## Supplementary Information

Below is the link to the electronic supplementary material.

ESM 1 Supplementary Material 1 (DOCX 17.3 KB)

ESM 2 Supplementary Material 2 (DOCX 176 KB)

ESM 3 Supplementary Material 3 (DOCX 179 KB)

## Data Availability

Available upon reasonable request **.**

## Abbreviations

aSAH: Aneurysmal Subarachnoid Hemorrhage
ANN: Artificial Neural Network
AUC-ROC: Area Under the Receiver Operating Characteristic Curve
CSF: Cerebrospinal Fluid
DL: Deep Learning
DOR: Diagnostic Odds Ratio
GCS: Glasgow Coma Scale
ICU: Intensive Care Unit
LR: Logistic Regression
ML: Machine Learning
NB: Naïve Bayes
NPV: Negative Predictive Value
OR: Odds Ratio
PPV: Positive Predictive Value
RF: Random Forest
ROC: Receiver Operating Characteristic
SHAP: SHapley Additive exPlanations
SVM: Support Vector Machine
WFNS: World Federation of Neurosurgical Societies grade
XGB: Extreme Gradient Boosting

## References

[1] Da Silva IRF, Gomes JA, Wachsman A, De Freitas GR, Provencio JJ (2017) Hematologic counts as predictors of delayed cerebral ischemia after aneurysmal subarachnoid hemorrhage. J Crit Care 37:126–129 27718411 10.1016/j.jcrc.2016.09.011 PMC5595061

[2] Dumont TM (2016) Prospective assessment of a symptomatic cerebral vasospasm predictive neural network model. World Neurosurg 94:126–130 27392898 10.1016/j.wneu.2016.06.110

[3] Dumont TM, Rughani AI, Tranmer BI (2011) Prediction of symptomatic cerebral vasospasm after aneurysmal subarachnoid hemorrhage with an artificial neural network: feasibility and comparison with logistic regression models. World Neurosurg 75(1):57–63 (**discussion 25–28**) 21492664 10.1016/j.wneu.2010.07.007

[4] Kim KH, Koo H-W, Lee B-J, Sohn M-J (2021) Analysis of risk factors correlated with angiographic vasospasm in patients with aneurysmal subarachnoid hemorrhage using explainable predictive modeling. J Clin Neurosci 91:334–342 34373049 10.1016/j.jocn.2021.07.028

[5] Kim KH, Lee B-J, Koo H-W (2023) Effect of cilostazol on delayed cerebral infarction in aneurysmal subarachnoid hemorrhage using explainable predictive modeling. Bioengineering 10(7):797 37508824 10.3390/bioengineering10070797 PMC10376257

[6] Lucke-Wold B, Dodd W, Motwani K et al (2022) Investigation and modulation of interleukin-6 following subarachnoid hemorrhage: targeting inflammatory activation for cerebral vasospasm. J Neuroinflammation 19(1):228 36114540 10.1186/s12974-022-02592-x PMC9479230

[7] Maroufi SF, Pachón-Londoño MJ, Ghoche M, Nguyen BA, Turcotte EL, Wang Z, Patra DP, Olson V, Halpin BS, Bathini AR, Meyer JH, Krishna C, Charbel FT, Morcos JJ, Batjer HH, Bendok BR (2025) Machine learning–based rupture risk prediction for intracranial aneurysms: a systematic review and meta-analysis. Neurosurgery 97(5):1072–1082. 10.1227/neu.0000000000003531 40444989 10.1227/neu.0000000000003531

[8] Mavridis I, Pyrgelis E-S, Agapiou E, Assi J (2024) Vasospasm in pediatric subarachnoid hemorrhage. CNS Neurol Disord Drug Targets 23(11):1303–1307 38013445 10.2174/0118715273274147231104160152

[9] Mohammadzadeh I, Niroomand B, Eini P, Khaledian H, Choubineh T, Luzzi S (2025) Leveraging machine learning algorithms to forecast delayed cerebral ischemia following subarachnoid hemorrhage: a systematic review and meta-analysis of 5,115 participants. Neurosurg Rev 48(1):26 39775123 10.1007/s10143-024-03175-5

[10] Page MJ, McKenzie JE, Bossuyt PM et al (2021) The PRISMA 2020 statement: an updated guideline for reporting systematic reviews. BMJ 372:n71 10.1136/bmj.n71 PMC8005924 33782057

[11] Palermo M, D’Arrigo S, Albanese A, Sturiale CL (2025) Enhancing cerebrospinal fluid clearance in subarachnoid hemorrhage: A systematic review of combined ventricular and lumbar drainage. J Clin Neurosci 139:111442 40614635 10.1016/j.jocn.2025.111442

[12] Palmisciano P, Hoz SS, Johnson MD, Forbes JA, Prestigiacomo CJ, Zuccarello M, Andaluz N (2023) External validation of an extreme gradient boosting model for prediction of delayed cerebral ischemia after aneurysmal subarachnoid hemorrhage. World Neurosurg 175:e108–e114 36914029 10.1016/j.wneu.2023.03.036

[13] Roederer A, Holmes JH, Smith MJ, Lee I, Park S (2014) Prediction of significant vasospasm in aneurysmal subarachnoid hemorrhage using automated data. Neurocrit Care 21(3):444–450 24715326 10.1007/s12028-014-9976-9

[14] Santana LS, Diniz JBC, Rabelo NN, Teixeira MJ, Figueiredo EG, Telles JPM (2024) Machine learning algorithms to predict delayed cerebral ischemia after subarachnoid hemorrhage: a systematic review and meta-analysis. Neurocrit Care 40(3):1171–1181 37667079 10.1007/s12028-023-01832-z

[15] Scherer M, Jung J-O, Cordes J, Wessels L, Younsi A, Schönenberger S, Möhlenbruch MA, Maier-Hein K, Unterberg A, Zweckberger K (2020) Association of cerebrospinal fluid volume with cerebral vasospasm after aneurysmal subarachnoid hemorrhage: a retrospective volumetric analysis. Neurocrit Care 33(1):152–164 31773545 10.1007/s12028-019-00878-2

[16] Skoch J, Tahir R, Abruzzo T, Taylor JM, Zuccarello M, Vadivelu S (2017) Predicting symptomatic cerebral vasospasm after aneurysmal subarachnoid hemorrhage with an artificial neural network in a pediatric population. Childs Nerv Syst 33(12):2153–2157 28852853 10.1007/s00381-017-3573-0

[17] Street JS, Pandit AS, Toma AK (2023) Predicting vasospasm risk using first presentation aneurysmal subarachnoid hemorrhage volume: A semi-automated CT image segmentation analysis using ITK-SNAP. PLoS ONE 18(6):e0286485 37262041 10.1371/journal.pone.0286485 PMC10234558

[18] Urbanos G, Castaño-León AM, Maldonado-Luna M, Salvador E, Ramos A, Lechuga C, Sanz C, Juárez E, Lagares A (2025) Comprehensive predictive modeling in subarachnoid hemorrhage: integrating radiomics and clinical variables. Neurosurg Rev 48(1):528 40553205 10.1007/s10143-025-03679-8 PMC12187877

[19] Van Der Steen WE, Marquering HA, Boers AMM et al (2019) Predicting delayed cerebral ischemia with quantified aneurysmal subarachnoid blood volume. World Neurosurg 130:e613–e619 31260850 10.1016/j.wneu.2019.06.170

[20] Vergouwen MDI, Ilodigwe D, Macdonald RL (2011) Cerebral infarction after subarachnoid hemorrhage contributes to poor outcome by vasospasm-dependent and -independent effects. Stroke 42(4):924–929 21311062 10.1161/STROKEAHA.110.597914

[21] Wolff RF, Moons KGM, Riley RD, Whiting PF, Westwood M, Collins GS, Reitsma JB, Kleijnen J, Mallett S, PROBAST Group† (2019) PROBAST: a tool to assess the risk of bias and applicability of prediction model studies. Ann Intern Med 170(1):51–58 10.7326/M18-1376 30596875

[22] Yang C, Luo F, Chen X, Deng Y, Lan M (2025) Association between multiple serum biomarkers and cerebral vasospasm in patients with aneurysmal subarachnoid hemorrhage. Front Neurol. 10.3389/fneur.2025.1592390 40538654 10.3389/fneur.2025.1592390 PMC12176561

[23] Yang J, Zhang X, Yang X, Wang J, You C, Ma L, Guan J (2025) Comparative effectiveness of different surgical timings on neurological outcomes for cranioplasty: protocol for a prospective non-randomized controlled trial. PLoS ONE 20(3):e0318841 40063640 10.1371/journal.pone.0318841 PMC11892811

[24] Zarrin DA, Suri A, McCarthy K, Gaonkar B, Wilson BR, Colby GP, Freundlich RE, Gabel E (2024) Machine learning predicts cerebral vasospasm in patients with subarachnoid haemorrhage. EBioMedicine. 10.1016/j.ebiom.2024.105206 38901147 10.1016/j.ebiom.2024.105206 PMC11245940

[25] Zhang H, Zou P, Luo P, Jiang X (2025) Machine learning for the early prediction of delayed cerebral ischemia in patients with subarachnoid hemorrhage: systematic review and meta-analysis. J Med Internet Res 27:e54121 39832368 10.2196/54121 PMC11791451

