# Supplementary material for: Machine learning models for predicting vasospasm following ruptured intracranial aneurysms: a systematic review and meta-analysis
Source: Acta Neurochir (Wien). 2025 Dec 3;167(1):314. doi: 10.1007/s00701-025-06725-y (PMC12678459; doi:10.1007/s00701-025-06725-y)
Supplement: Supplementary file 1 — Supplementary Material 1 (DOCX 17.3 KB) [file 701_2025_6725_MOESM1_ESM.docx]

**Table 1 (Supplementary material):** PROBAST (Prediction model Risk of Bias Assessment Tool) Review Items

Possible answers: Yes (Y), probably Y (PY), probably no (PN), No (N), or No information (NI).

| Domain | Signaling Question |  |
| --- | --- | --- |
| 1. Participants | 1.1 Were appropriate data sources used, e.g., cohort, or nested case-control study data? | Y |
|  | 1.2 Were all inclusions and exclusions of participants appropriate? | Y |
| 2. Predictors | 2.1 Were predictors defined and assessed in a similar way for all participants? | Y |
|  | 2.2 Were predictor assessments made without knowledge of outcome data? | N |
|  | 2.3 Are all predictors available at the time the model is intended to be used? | Y |
| 3. Outcome | 3.1 Was the outcome determined appropriately? | Y |
|  | 3.2 Was a prespecified or standard outcome definition used? | PY |
|  | 3.3 Were predictors excluded from the outcome definition? | NI |
|  | 3.4 Was the outcome defined and determined in a similar way for all participants? | Y |
|  | 3.5 Was the outcome determined without knowledge of predictor information? | Y |
|  | 3.6 Was the time interval between predictor assessment and outcome determination appropriate? | Y |
| 4. Analysis | 4.1 Were there a reasonable number of participants with the outcome? | PY |
|  | 4.2 Were continuous and categorical predictors handled appropriately? | PY |
|  | 4.3 Were all enrolled participants included in the analysis? | PY |
|  | 4.4 Were participants with missing data handled appropriately? | Y |
|  | 4.5 Was selection of predictors based on univariable analysis avoided? | N |
|  | 4.6 Were complexities in the data (e.g., censoring, competing risks, sampling of control participants accounted for appropriately? | Y |
|  | 4.7 Were relevant model performance measures evaluated appropriately? | Y |
|  | 4.8 Were model overfitting and optimism in model performance accounted for? | Y |
|  | 4.9 Do predictors and their assigned weights in the final model correspond to the results from the reported multivariable analysis? | Y |

All articles included in this review reported the same result in the PROBAST risk of bias questionnaire
