# Supplementary material for: Machine learning models for predicting vasospasm following ruptured intracranial aneurysms: a systematic review and meta-analysis
Source: Acta Neurochir (Wien). 2025 Dec 3;167(1):314. doi: 10.1007/s00701-025-06725-y (PMC12678459; doi:10.1007/s00701-025-06725-y)
Supplement: Supplementary file 2 — Supplementary Material 2 (DOCX 176 KB) [file 701_2025_6725_MOESM2_ESM.docx]

**Table 2 - Suppl. Material:** two-way ANOVA per algorithm type.

**ALGORITHM - ACCURACY**

| *ANOVA - accuracy* | | | | | | | | | | | |
| --- | --- | --- | --- | --- | --- | --- | --- | --- | --- | --- | --- |
| Cases | | Sum of Squares | | df | | Mean Square | | F | | p | |
| Algorithm |  | 6272.063 |  | 3 |  | 2090.688 |  | 2.739 |  | 0.070 |  |
| Residuals |  | 15264.117 |  | 20 |  | 763.206 |  |  |  |  |  |
|  | | | | | | | | | | | |
| *Note.*  Type III Sum of Squares | | | | | | | | | | | |

**Descriptives**

| *Descriptives - accuracy* | | | | | | | | | | | |
| --- | --- | --- | --- | --- | --- | --- | --- | --- | --- | --- | --- |
| Algorithm | | N | | Mean | | SD | | SE | | Coefficient of variation | |
| Deep Learning |  | 9 |  | 90.589 |  | 5.571 |  | 1.857 |  | 0.062 |  |
| Ensemble Methods |  | 6 |  | 51.183 |  | 41.287 |  | 16.856 |  | 0.807 |  |
| Regression Methods |  | 7 |  | 64.371 |  | 32.739 |  | 12.374 |  | 0.509 |  |
| SVM Model |  | 2 |  | 80.850 |  | 7.849 |  | 5.550 |  | 0.097 |  |
|  | | | | | | | | | | | |


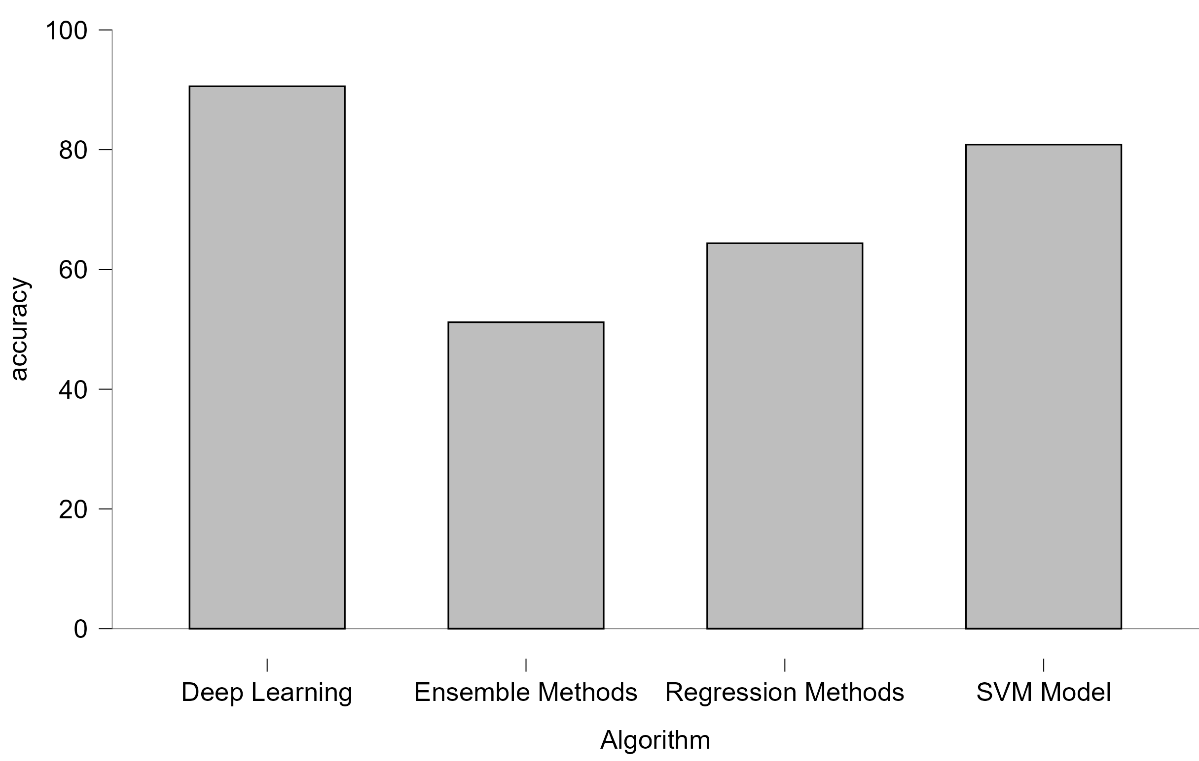


**Post Hoc Tests**

**Standard**

| *Post Hoc Comparisons - Algorithm* | | | | | | | | | | | | | |
| --- | --- | --- | --- | --- | --- | --- | --- | --- | --- | --- | --- | --- | --- |
|  | |  | | Mean Difference | | SE | | df | | t | | p_bonf_ | |
| Deep Learning |  | Ensemble Methods |  | 39.406 |  | 14.560 |  | 20 |  | 2.706 |  | 0.082 |  |
|  |  | Regression Methods |  | 26.217 |  | 13.922 |  | 20 |  | 1.883 |  | 0.446 |  |
|  |  | SVM Model |  | 9.739 |  | 21.596 |  | 20 |  | 0.451 |  | 1.000 |  |
| Ensemble Methods |  | Regression Methods |  | -13.188 |  | 15.370 |  | 20 |  | -0.858 |  | 1.000 |  |
|  |  | SVM Model |  | -29.667 |  | 22.557 |  | 20 |  | -1.315 |  | 1.000 |  |
| Regression Methods |  | SVM Model |  | -16.479 |  | 22.150 |  | 20 |  | -0.744 |  | 1.000 |  |
|  | | | | | | | | | | | | | |
| *Note.*  P-value adjusted for comparing a family of 6 estimates. | | | | | | | | | | | | | |

**Marginal Means**

| *Marginal Means - Algorithm* | | | | | | | | | |
| --- | --- | --- | --- | --- | --- | --- | --- | --- | --- |
|  | | | | 95% CI for Mean Difference | | | |  | |
| Algorithm | | Marginal Mean | | Lower | | Upper | | SE | |
| Deep Learning |  | 90.589 |  | 71.380 |  | 109.798 |  | 9.209 |  |
| Ensemble Methods |  | 51.183 |  | 27.657 |  | 74.710 |  | 11.278 |  |
| Regression Methods |  | 64.371 |  | 42.590 |  | 86.152 |  | 10.442 |  |
| SVM Model |  | 80.850 |  | 40.101 |  | 121.599 |  | 19.535 |  |
|  | | | | | | | | | |

**ALGORITHM - SENSITIVITY**

| *ANOVA - sensitivity* | | | | | | | | | | | |
| --- | --- | --- | --- | --- | --- | --- | --- | --- | --- | --- | --- |
| Cases | | Sum of Squares | | df | | Mean Square | | F | | p | |
| Algorithm |  | 4268.955 |  | 3 |  | 1422.985 |  | 6.864 |  | 0.003 |  |
| Residuals |  | 3316.917 |  | 16 |  | 207.307 |  |  |  |  |  |
|  | | | | | | | | | | | |
| *Note.*  Type III Sum of Squares | | | | | | | | | | | |

**Bar plots**


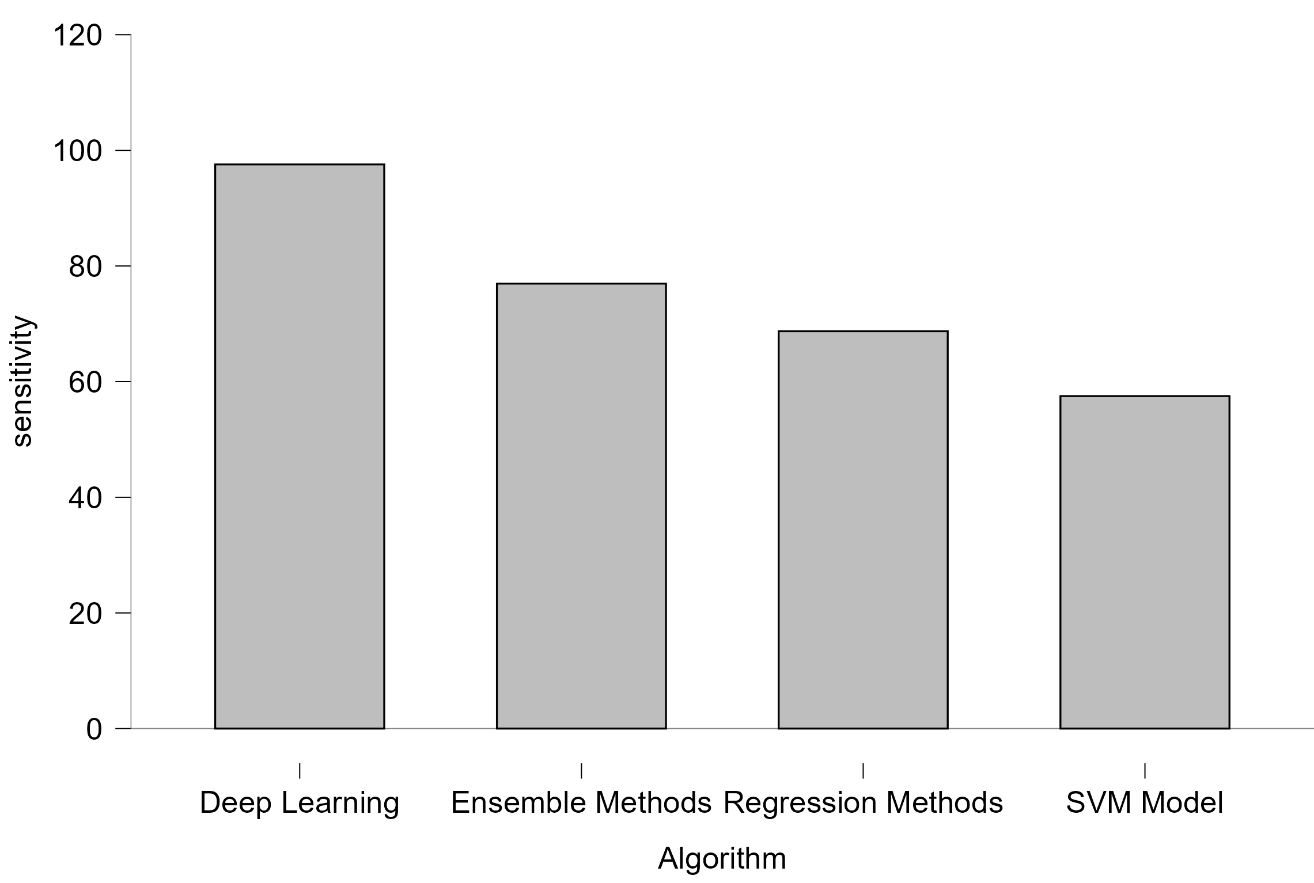


**Post Hoc Tests**

**Standard**

| *Post Hoc Comparisons - Algorithm* | | | | | | | | | | | | | |
| --- | --- | --- | --- | --- | --- | --- | --- | --- | --- | --- | --- | --- | --- |
|  | |  | | Mean Difference | | SE | | df | | t | | p_bonf_ | |
| Deep Learning |  | Ensemble Methods |  | 20.613 |  | 8.817 |  | 16 |  | 2.338 |  | 0.196 |  |
|  |  | Regression Methods |  | 28.846 |  | 7.776 |  | 16 |  | 3.710 |  | 0.011 |  |
|  |  | SVM Model |  | 40.063 |  | 11.383 |  | 16 |  | 3.520 |  | 0.017 |  |
| Ensemble Methods |  | Regression Methods |  | 8.233 |  | 9.294 |  | 16 |  | 0.886 |  | 1.000 |  |
|  |  | SVM Model |  | 19.450 |  | 12.469 |  | 16 |  | 1.560 |  | 0.830 |  |
| Regression Methods |  | SVM Model |  | 11.217 |  | 11.756 |  | 16 |  | 0.954 |  | 1.000 |  |
|  | | | | | | | | | | | | | |
| *Note.*  P-value adjusted for comparing a family of 6 estimates. | | | | | | | | | | | | | |

**Marginal Means**

| *Marginal Means - Algorithm* | | | | | | | | | |
| --- | --- | --- | --- | --- | --- | --- | --- | --- | --- |
|  | | | | 95% CI for Mean Difference | | | |  | |
| Algorithm | | Marginal Mean | | Lower | | Upper | | SE | |
| Deep Learning |  | 97.563 |  | 86.771 |  | 108.354 |  | 5.091 |  |
| Ensemble Methods |  | 76.950 |  | 61.689 |  | 92.211 |  | 7.199 |  |
| Regression Methods |  | 68.717 |  | 56.256 |  | 81.178 |  | 5.878 |  |
| SVM Model |  | 57.500 |  | 35.917 |  | 79.083 |  | 10.181 |  |
|  | | | | | | | | | |

**ALGORITHM - SPECIFICITY**

| *ANOVA - specificity* | | | | | | | | | | | |
| --- | --- | --- | --- | --- | --- | --- | --- | --- | --- | --- | --- |
| Cases | | Sum of Squares | | df | | Mean Square | | F | | p | |
| Algorithm |  | 1117.822 |  | 3 |  | 372.607 |  | 1.487 |  | 0.256 |  |
| Residuals |  | 4009.816 |  | 16 |  | 250.613 |  |  |  |  |  |
|  | | | | | | | | | | | |
| *Note.*  Type III Sum of Squares | | | | | | | | | | | |

**Bar plots**


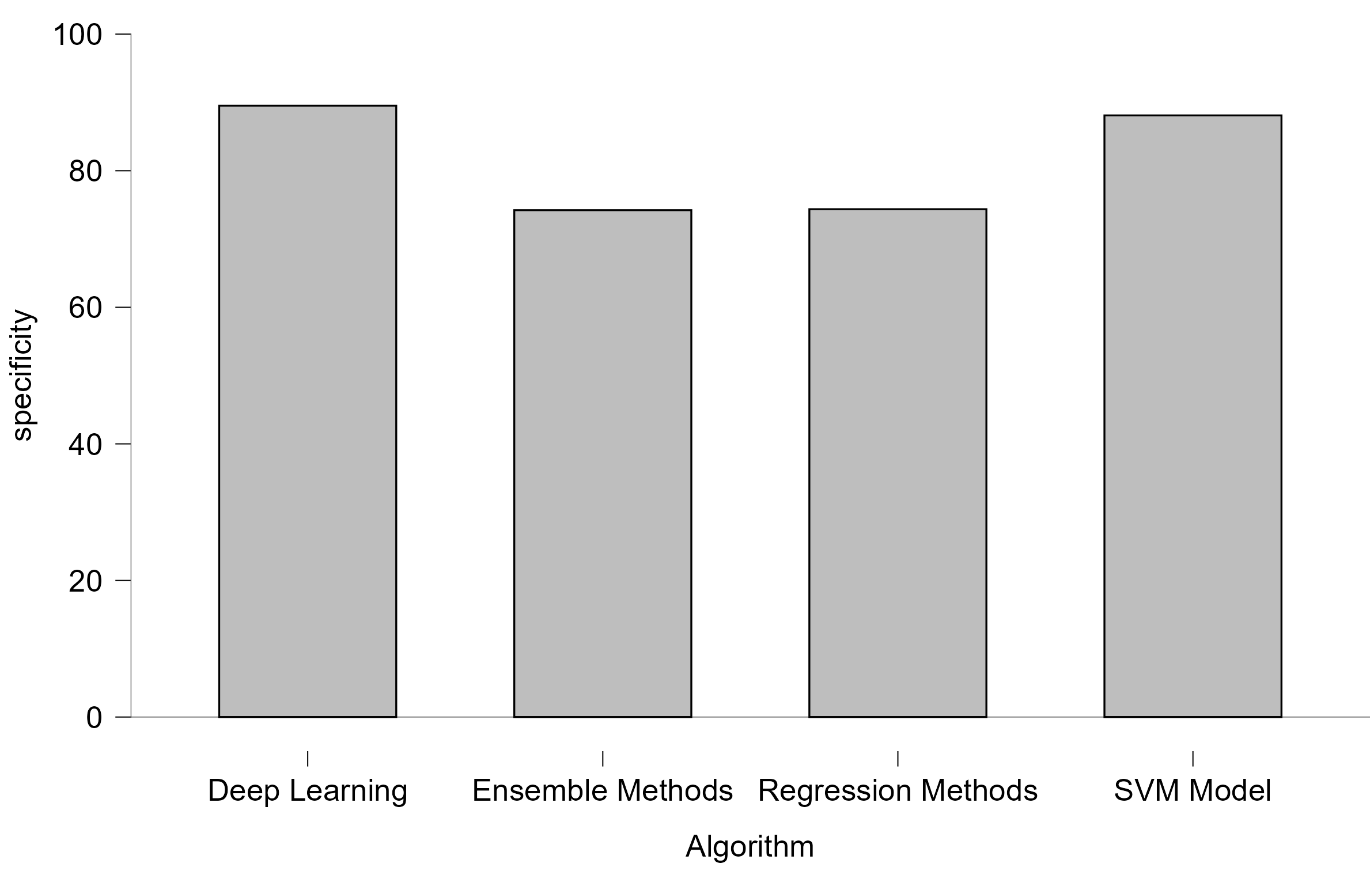


**Post Hoc Tests**

**Standard**

| *Post Hoc Comparisons - Algorithm* | | | | | | | | | | | | | |
| --- | --- | --- | --- | --- | --- | --- | --- | --- | --- | --- | --- | --- | --- |
|  | |  | | Mean Difference | | SE | | df | | t | | p_bonf_ | |
| Deep Learning |  | Ensemble Methods |  | 15.300 |  | 9.694 |  | 16 |  | 1.578 |  | 0.804 |  |
|  |  | Regression Methods |  | 15.158 |  | 8.550 |  | 16 |  | 1.773 |  | 0.572 |  |
|  |  | SVM Model |  | 1.425 |  | 12.515 |  | 16 |  | 0.114 |  | 1.000 |  |
| Ensemble Methods |  | Regression Methods |  | -0.142 |  | 10.219 |  | 16 |  | -0.014 |  | 1.000 |  |
|  |  | SVM Model |  | -13.875 |  | 13.710 |  | 16 |  | -1.012 |  | 1.000 |  |
| Regression Methods |  | SVM Model |  | -13.733 |  | 12.926 |  | 16 |  | -1.062 |  | 1.000 |  |
|  | | | | | | | | | | | | | |
| *Note.*  P-value adjusted for comparing a family of 6 estimates. | | | | | | | | | | | | | |

**Marginal Means**

| *Marginal Means - Algorithm* | | | | | | | | | |
| --- | --- | --- | --- | --- | --- | --- | --- | --- | --- |
|  | | | | 95% CI for Mean Difference | | | |  | |
| Algorithm | | Marginal Mean | | Lower | | Upper | | SE | |
| Deep Learning |  | 89.525 |  | 77.660 |  | 101.390 |  | 5.597 |  |
| Ensemble Methods |  | 74.225 |  | 57.445 |  | 91.005 |  | 7.915 |  |
| Regression Methods |  | 74.367 |  | 60.666 |  | 88.067 |  | 6.463 |  |
| SVM Model |  | 88.100 |  | 64.370 |  | 111.830 |  | 11.194 |  |
|  | | | | | | | | | |

**ALGORITHM - AUC**

| *ANOVA - AUC-ROC* | | | | | | | | | | | |
| --- | --- | --- | --- | --- | --- | --- | --- | --- | --- | --- | --- |
| Cases | | Sum of Squares | | df | | Mean Square | | F | | p | |
| Algorithm |  | 724.135 |  | 2 |  | 362.067 |  | 0.629 |  | 0.551 |  |
| Residuals |  | 6333.101 |  | 11 |  | 575.736 |  |  |  |  |  |
|  | | | | | | | | | | | |
| *Note.*  Type III Sum of Squares | | | | | | | | | | | |

**Bar plots**


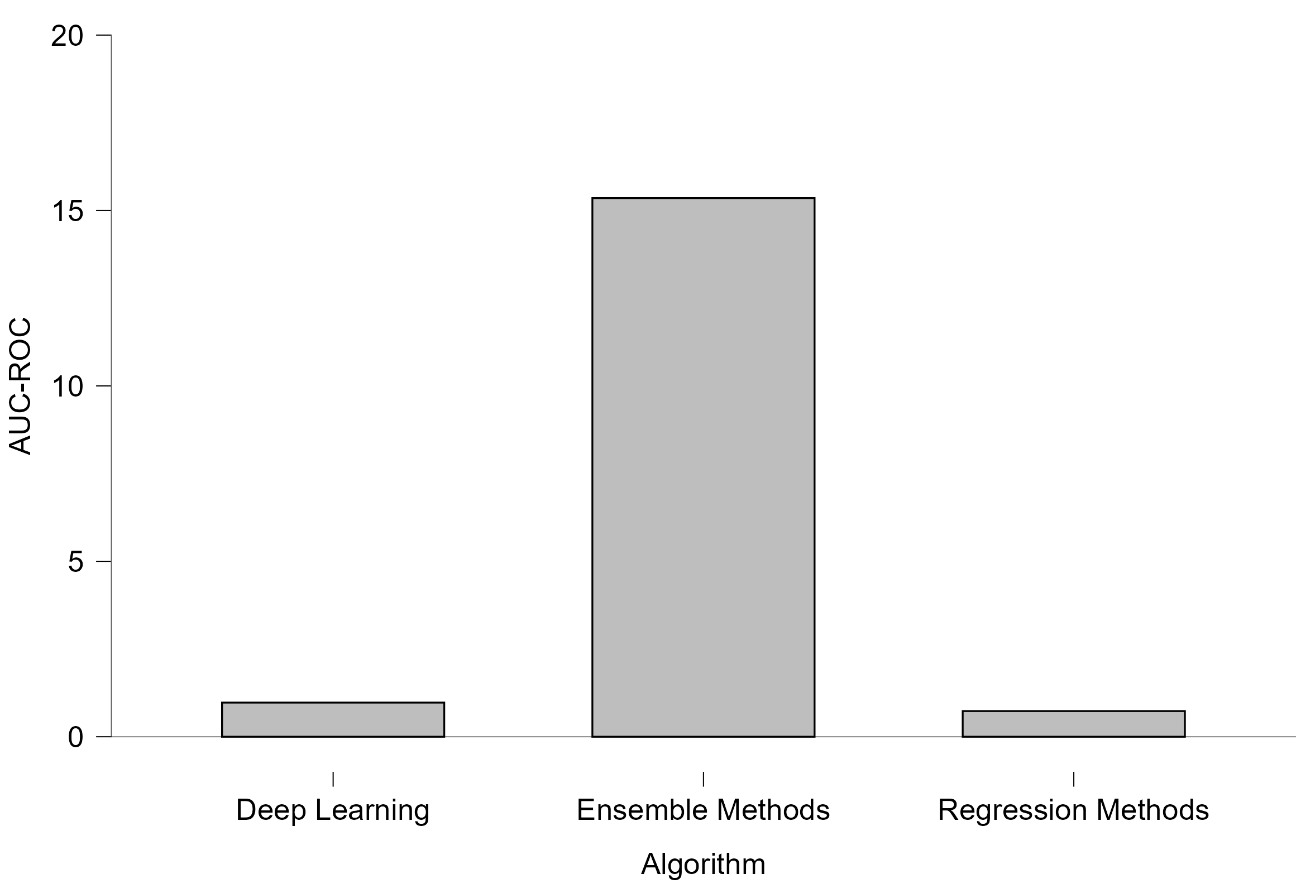


**Post Hoc Tests**

**Standard**

| *Post Hoc Comparisons - Algorithm* | | | | | | | | | | | | | |
| --- | --- | --- | --- | --- | --- | --- | --- | --- | --- | --- | --- | --- | --- |
|  | |  | | Mean Difference | | SE | | df | | t | | p_bonf_ | |
| Deep Learning |  | Ensemble Methods |  | -14.381 |  | 16.967 |  | 11 |  | -0.848 |  | 1.000 |  |
|  |  | Regression Methods |  | 0.241 |  | 17.523 |  | 11 |  | 0.014 |  | 1.000 |  |
| Ensemble Methods |  | Regression Methods |  | 14.622 |  | 14.529 |  | 11 |  | 1.006 |  | 1.000 |  |
|  | | | | | | | | | | | | | |
| *Note.*  P-value adjusted for comparing a family of 3 estimates. | | | | | | | | | | | | | |

**Marginal Means**

| *Marginal Means - Algorithm* | | | | | | | | | |
| --- | --- | --- | --- | --- | --- | --- | --- | --- | --- |
|  | | | | 95% CI for Mean Difference | | | |  | |
| Algorithm | | Marginal Mean | | Lower | | Upper | | SE | |
| Deep Learning |  | 0.973 |  | -29.517 |  | 31.464 |  | 13.853 |  |
| Ensemble Methods |  | 15.354 |  | -6.206 |  | 36.915 |  | 9.796 |  |
| Regression Methods |  | 0.732 |  | -22.886 |  | 24.350 |  | 10.731 |  |
|  | | | | | | | | | |

**ALGORITHM - PPV**

| *ANOVA - PPV* | | | | | | | | | | | |
| --- | --- | --- | --- | --- | --- | --- | --- | --- | --- | --- | --- |
| Cases | | Sum of Squares | | df | | Mean Square | | F | | p | |
| Algorithm |  | 15750.918 |  | 3 |  | 5250.306 |  | 6.877 |  | 0.003 |  |
| Residuals |  | 12978.602 |  | 17 |  | 763.447 |  |  |  |  |  |
|  | | | | | | | | | | | |
| *Note.*  Type III Sum of Squares | | | | | | | | | | | |

**Bar plots**


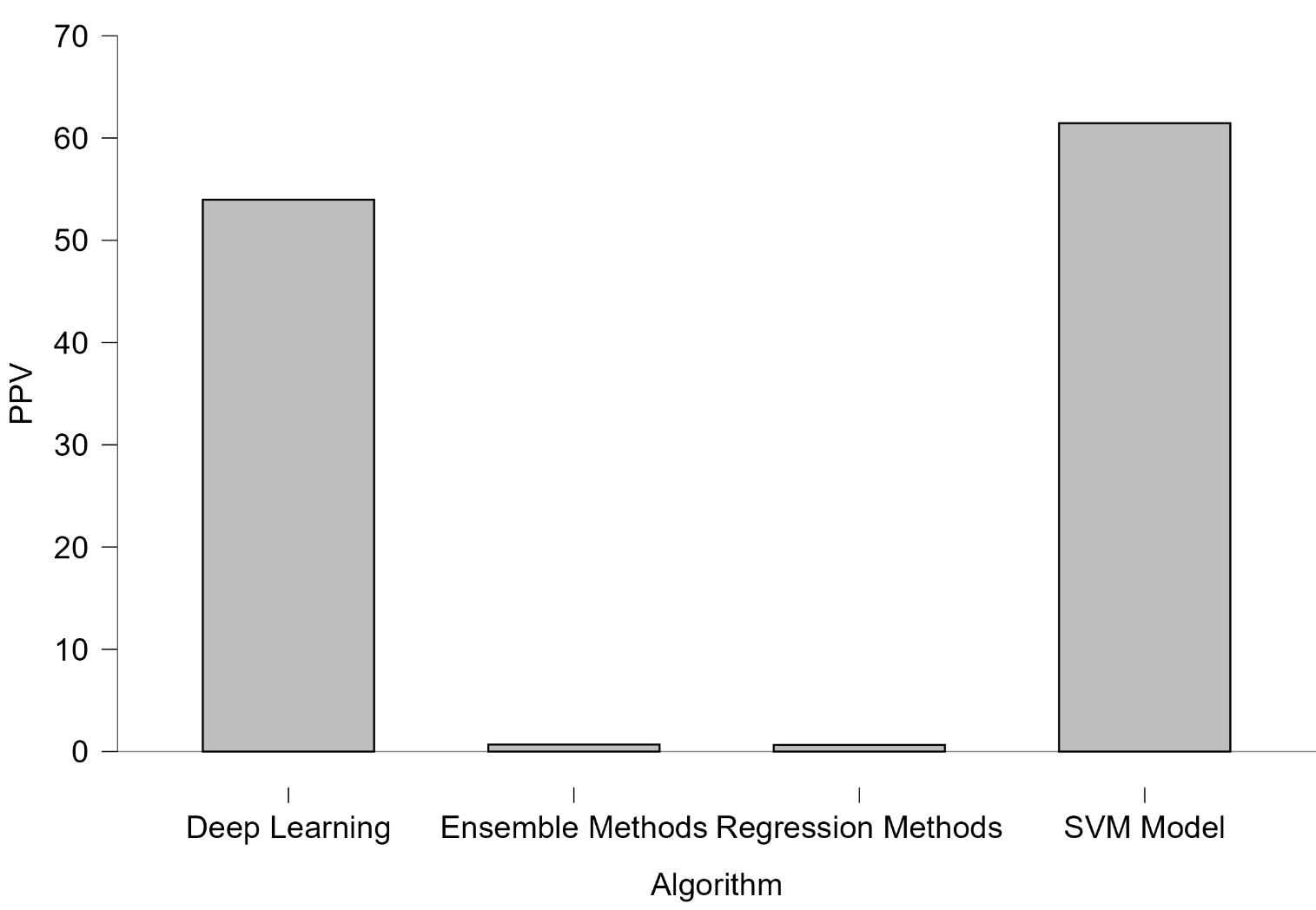


**Post Hoc Tests**

**Standard**

| *Post Hoc Comparisons - Algorithm* | | | | | | | | | | | | | |
| --- | --- | --- | --- | --- | --- | --- | --- | --- | --- | --- | --- | --- | --- |
|  | |  | | Mean Difference | | SE | | df | | t | | p_bonf_ | |
| Deep Learning |  | Ensemble Methods |  | 53.289 |  | 16.604 |  | 17 |  | 3.209 |  | 0.031 |  |
|  |  | Regression Methods |  | 53.336 |  | 14.563 |  | 17 |  | 3.663 |  | 0.012 |  |
|  |  | SVM Model |  | -7.476 |  | 21.600 |  | 17 |  | -0.346 |  | 1.000 |  |
| Ensemble Methods |  | Regression Methods |  | 0.047 |  | 17.835 |  | 17 |  | 0.003 |  | 1.000 |  |
|  |  | SVM Model |  | -60.765 |  | 23.929 |  | 17 |  | -2.539 |  | 0.127 |  |
| Regression Methods |  | SVM Model |  | -60.812 |  | 22.560 |  | 17 |  | -2.696 |  | 0.092 |  |
|  | | | | | | | | | | | | | |
| *Note.*  P-value adjusted for comparing a family of 6 estimates. | | | | | | | | | | | | | |

**Marginal Means**

| *Marginal Means - Algorithm* | | | | | | | | | |
| --- | --- | --- | --- | --- | --- | --- | --- | --- | --- |
|  | | | | 95% CI for Mean Difference | | | |  | |
| Algorithm | | Marginal Mean | | Lower | | Upper | | SE | |
| Deep Learning |  | 53.974 |  | 34.543 |  | 73.406 |  | 9.210 |  |
| Ensemble Methods |  | 0.685 |  | -28.463 |  | 29.833 |  | 13.815 |  |
| Regression Methods |  | 0.638 |  | -23.161 |  | 24.437 |  | 11.280 |  |
| SVM Model |  | 61.450 |  | 20.229 |  | 102.671 |  | 19.538 |  |
|  | | | | | | | | | |

**ALGORITHM - NPV**

| *ANOVA - NPV* | | | | | | | | | | | |
| --- | --- | --- | --- | --- | --- | --- | --- | --- | --- | --- | --- |
| Cases | | Sum of Squares | | df | | Mean Square | | F | | p | |
| Algorithm |  | 22240.401 |  | 3 |  | 7413.467 |  | 6.712 |  | 0.004 |  |
| Residuals |  | 17672.926 |  | 16 |  | 1104.558 |  |  |  |  |  |
|  | | | | | | | | | | | |
| *Note.*  Type III Sum of Squares | | | | | | | | | | | |

**Bar plots**


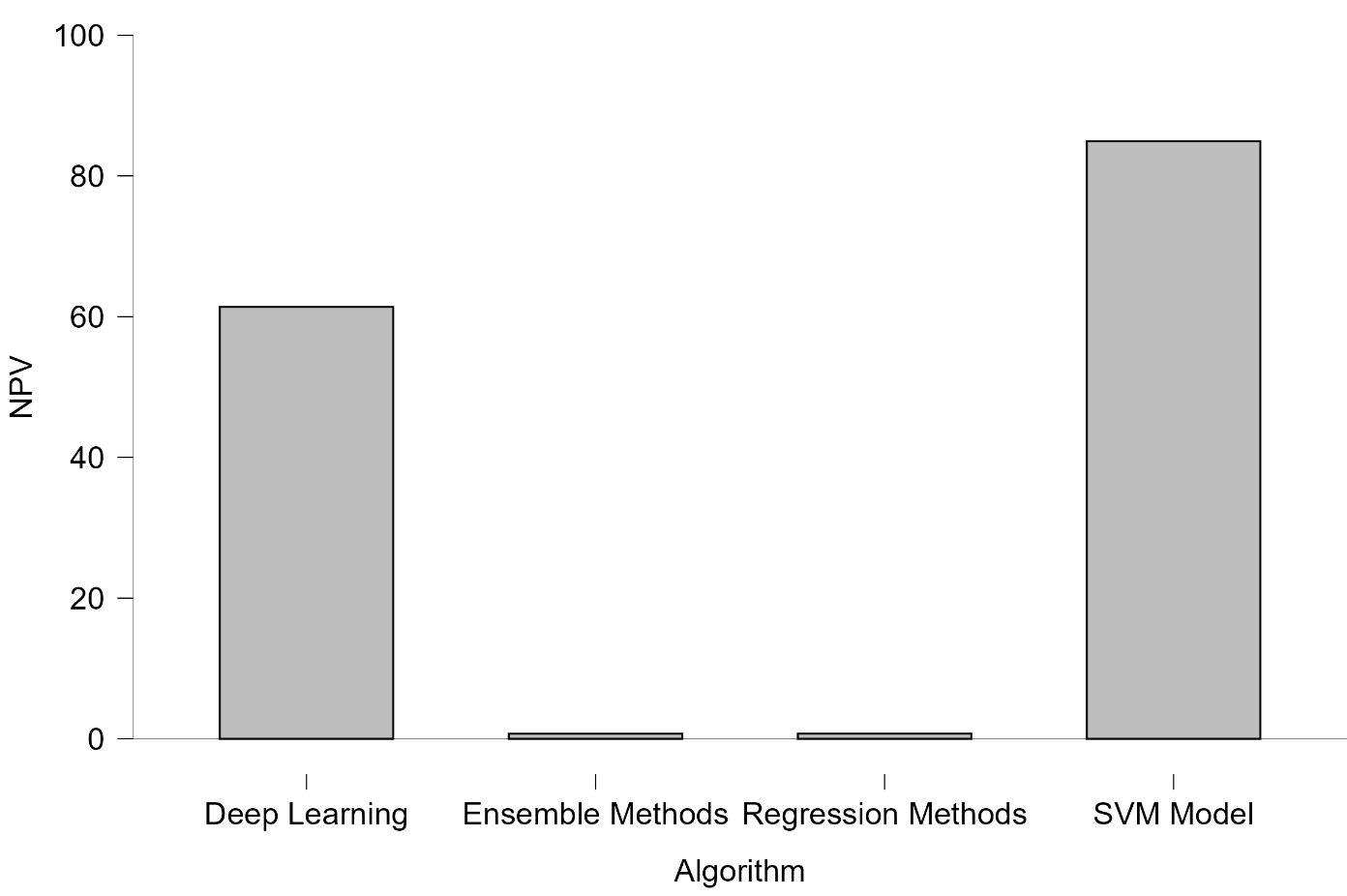


**Post Hoc Tests**

**Standard**

| *Post Hoc Comparisons - Algorithm* | | | | | | | | | | | | | |
| --- | --- | --- | --- | --- | --- | --- | --- | --- | --- | --- | --- | --- | --- |
|  | |  | | Mean Difference | | SE | | df | | t | | p_bonf_ | |
| Deep Learning |  | Ensemble Methods |  | 60.640 |  | 20.352 |  | 16 |  | 2.980 |  | 0.053 |  |
|  |  | Regression Methods |  | 60.627 |  | 17.949 |  | 16 |  | 3.378 |  | 0.023 |  |
|  |  | SVM Model |  | -23.575 |  | 26.274 |  | 16 |  | -0.897 |  | 1.000 |  |
| Ensemble Methods |  | Regression Methods |  | -0.013 |  | 21.453 |  | 16 |  | -6.215×10^-4^ |  | 1.000 |  |
|  |  | SVM Model |  | -84.215 |  | 28.782 |  | 16 |  | -2.926 |  | 0.059 |  |
| Regression Methods |  | SVM Model |  | -84.202 |  | 27.136 |  | 16 |  | -3.103 |  | 0.041 |  |
|  | | | | | | | | | | | | | |
| *Note.*  P-value adjusted for comparing a family of 6 estimates. | | | | | | | | | | | | | |

**Marginal Means**

| *Marginal Means - Algorithm* | | | | | | | | | |
| --- | --- | --- | --- | --- | --- | --- | --- | --- | --- |
|  | | | | 95% CI for Mean Difference | | | |  | |
| Algorithm | | Marginal Mean | | Lower | | Upper | | SE | |
| Deep Learning |  | 61.375 |  | 36.465 |  | 86.285 |  | 11.750 |  |
| Ensemble Methods |  | 0.735 |  | -34.492 |  | 35.962 |  | 16.617 |  |
| Regression Methods |  | 0.748 |  | -28.015 |  | 29.511 |  | 13.568 |  |
| SVM Model |  | 84.950 |  | 35.131 |  | 134.769 |  | 23.501 |  |
|  | | | | | | | | | |
