# Supplementary material for: Machine learning models for predicting vasospasm following ruptured intracranial aneurysms: a systematic review and meta-analysis
Source: Acta Neurochir (Wien). 2025 Dec 3;167(1):314. doi: 10.1007/s00701-025-06725-y (PMC12678459; doi:10.1007/s00701-025-06725-y)
Supplement: Supplementary file 3 — Supplementary Material 3 (DOCX 179 KB) [file 701_2025_6725_MOESM3_ESM.docx]

**Table 3 Suppl. Material:** Kruskal–Wallis Test Results: Comparison of Model Performance Metrics Across Algorithm Types and Cohorts

**COHORT X ALGORITHM ACCURACY**

| *Descriptives - accuracy* | | | | | | | | | | | | | |
| --- | --- | --- | --- | --- | --- | --- | --- | --- | --- | --- | --- | --- | --- |
| Cohort Type | | Algorithm | | N | | Mean | | SD | | SE | | Coefficient of variation | |
| TEST |  | Deep Learning |  | 4 |  | 87.600 |  | 6.015 |  | 3.007 |  | 0.069 |  |
|  |  | Ensemble Methods |  | 5 |  | 61.420 |  | 36.673 |  | 16.401 |  | 0.597 |  |
|  |  | Regression Methods |  | 6 |  | 67.083 |  | 34.992 |  | 14.285 |  | 0.522 |  |
|  |  | SVM Model |  | 1 |  | 86.400 |  |  |  |  |  |  |  |
| TRAIN |  | Deep Learning |  | 3 |  | 92.300 |  | 0.000 |  | 0.000 |  | 0.000 |  |
|  |  | Generative Models |  | 1 |  | 60.500 |  |  |  |  |  |  |  |
|  |  | Regression Methods |  | 1 |  | 48.100 |  |  |  |  |  |  |  |
|  |  | SVM Model |  | 1 |  | 75.300 |  |  |  |  |  |  |  |
| VALIDATION |  | Deep Learning |  | 2 |  | 94.000 |  | 8.485 |  | 6.000 |  | 0.090 |  |
|  |  | Ensemble Methods |  | 1 |  | 0.000 |  |  |  |  |  |  |  |
|  | | | | | | | | | | | | | |
| *Note.*  Some combinations of factors are not observed and hence omitted (5 out of 15 combinations are unobserved). | | | | | | | | | | | | | |

**Post Hoc Tests**

**Standard**

| *Post Hoc Comparisons - Cohort Type ✻ Algorithm* | | | | | | | | | | | | | |
| --- | --- | --- | --- | --- | --- | --- | --- | --- | --- | --- | --- | --- | --- |
|  | |  | | Mean Difference | | SE | | df | | t | | p_bonf_ | |
| TEST Deep Learning |  | TRAIN Deep Learning |  | -4.700 |  | 21.314 |  | 15 |  | -0.221 |  | 1.000 |  |
|  |  | VALIDATION Deep Learning |  | -6.400 |  | 24.168 |  | 15 |  | -0.265 |  | 1.000 |  |
|  |  | TEST Ensemble Methods |  | 26.180 |  | 18.721 |  | 15 |  | 1.398 |  | 1.000 |  |
|  |  | TRAIN Ensemble Methods |  |  |  |  |  |  |  |  |  |  |  |
|  |  | VALIDATION Ensemble Methods |  | 87.600 |  | 31.201 |  | 15 |  | 2.808 |  | 0.596 |  |
|  |  | TEST Generative Models |  |  |  |  |  |  |  |  |  |  |  |
|  |  | TRAIN Generative Models |  | 27.100 |  | 31.201 |  | 15 |  | 0.869 |  | 1.000 |  |
|  |  | VALIDATION Generative Models |  |  |  |  |  |  |  |  |  |  |  |
|  |  | TEST Regression Methods |  | 20.517 |  | 18.014 |  | 15 |  | 1.139 |  | 1.000 |  |
|  |  | TRAIN Regression Methods |  | 39.500 |  | 31.201 |  | 15 |  | 1.266 |  | 1.000 |  |
|  |  | VALIDATION Regression Methods |  |  |  |  |  |  |  |  |  |  |  |
|  |  | TEST SVM Model |  | 1.200 |  | 31.201 |  | 15 |  | 0.038 |  | 1.000 |  |
|  |  | TRAIN SVM Model |  | 12.300 |  | 31.201 |  | 15 |  | 0.394 |  | 1.000 |  |
|  |  | VALIDATION SVM Model |  |  |  |  |  |  |  |  |  |  |  |
| TRAIN Deep Learning |  | VALIDATION Deep Learning |  | -1.700 |  | 25.476 |  | 15 |  | -0.067 |  | 1.000 |  |
|  |  | TEST Ensemble Methods |  | 30.880 |  | 20.381 |  | 15 |  | 1.515 |  | 1.000 |  |
|  |  | TRAIN Ensemble Methods |  |  |  |  |  |  |  |  |  |  |  |
|  |  | VALIDATION Ensemble Methods |  | 92.300 |  | 32.224 |  | 15 |  | 2.864 |  | 0.532 |  |
|  |  | TEST Generative Models |  |  |  |  |  |  |  |  |  |  |  |
|  |  | TRAIN Generative Models |  | 31.800 |  | 32.224 |  | 15 |  | 0.987 |  | 1.000 |  |
|  |  | VALIDATION Generative Models |  |  |  |  |  |  |  |  |  |  |  |
|  |  | TEST Regression Methods |  | 25.217 |  | 19.733 |  | 15 |  | 1.278 |  | 1.000 |  |
|  |  | TRAIN Regression Methods |  | 44.200 |  | 32.224 |  | 15 |  | 1.372 |  | 1.000 |  |
|  |  | VALIDATION Regression Methods |  |  |  |  |  |  |  |  |  |  |  |
|  |  | TEST SVM Model |  | 5.900 |  | 32.224 |  | 15 |  | 0.183 |  | 1.000 |  |
|  |  | TRAIN SVM Model |  | 17.000 |  | 32.224 |  | 15 |  | 0.528 |  | 1.000 |  |
|  |  | VALIDATION SVM Model |  |  |  |  |  |  |  |  |  |  |  |
| VALIDATION Deep Learning |  | TEST Ensemble Methods |  | 32.580 |  | 23.349 |  | 15 |  | 1.395 |  | 1.000 |  |
|  |  | TRAIN Ensemble Methods |  |  |  |  |  |  |  |  |  |  |  |
|  |  | VALIDATION Ensemble Methods |  | 94.000 |  | 34.179 |  | 15 |  | 2.750 |  | 0.670 |  |
|  |  | TEST Generative Models |  |  |  |  |  |  |  |  |  |  |  |
|  |  | TRAIN Generative Models |  | 33.500 |  | 34.179 |  | 15 |  | 0.980 |  | 1.000 |  |
|  |  | VALIDATION Generative Models |  |  |  |  |  |  |  |  |  |  |  |
|  |  | TEST Regression Methods |  | 26.917 |  | 22.786 |  | 15 |  | 1.181 |  | 1.000 |  |
|  |  | TRAIN Regression Methods |  | 45.900 |  | 34.179 |  | 15 |  | 1.343 |  | 1.000 |  |
|  |  | VALIDATION Regression Methods |  |  |  |  |  |  |  |  |  |  |  |
|  |  | TEST SVM Model |  | 7.600 |  | 34.179 |  | 15 |  | 0.222 |  | 1.000 |  |
|  |  | TRAIN SVM Model |  | 18.700 |  | 34.179 |  | 15 |  | 0.547 |  | 1.000 |  |
|  |  | VALIDATION SVM Model |  |  |  |  |  |  |  |  |  |  |  |
| TEST Ensemble Methods |  | TRAIN Ensemble Methods |  |  |  |  |  |  |  |  |  |  |  |
|  |  | VALIDATION Ensemble Methods |  | 61.420 |  | 30.571 |  | 15 |  | 2.009 |  | 1.000 |  |
|  |  | TEST Generative Models |  |  |  |  |  |  |  |  |  |  |  |
|  |  | TRAIN Generative Models |  | 0.920 |  | 30.571 |  | 15 |  | 0.030 |  | 1.000 |  |
|  |  | VALIDATION Generative Models |  |  |  |  |  |  |  |  |  |  |  |
|  |  | TEST Regression Methods |  | -5.663 |  | 16.899 |  | 15 |  | -0.335 |  | 1.000 |  |
|  |  | TRAIN Regression Methods |  | 13.320 |  | 30.571 |  | 15 |  | 0.436 |  | 1.000 |  |
|  |  | VALIDATION Regression Methods |  |  |  |  |  |  |  |  |  |  |  |
|  |  | TEST SVM Model |  | -24.980 |  | 30.571 |  | 15 |  | -0.817 |  | 1.000 |  |
|  |  | TRAIN SVM Model |  | -13.880 |  | 30.571 |  | 15 |  | -0.454 |  | 1.000 |  |
|  |  | VALIDATION SVM Model |  |  |  |  |  |  |  |  |  |  |  |
| TRAIN Ensemble Methods |  | VALIDATION Ensemble Methods |  |  |  |  |  |  |  |  |  |  |  |
|  |  | TEST Generative Models |  |  |  |  |  |  |  |  |  |  |  |
|  |  | TRAIN Generative Models |  |  |  |  |  |  |  |  |  |  |  |
|  |  | VALIDATION Generative Models |  |  |  |  |  |  |  |  |  |  |  |
|  |  | TEST Regression Methods |  |  |  |  |  |  |  |  |  |  |  |
|  |  | TRAIN Regression Methods |  |  |  |  |  |  |  |  |  |  |  |
|  |  | VALIDATION Regression Methods |  |  |  |  |  |  |  |  |  |  |  |
|  |  | TEST SVM Model |  |  |  |  |  |  |  |  |  |  |  |
|  |  | TRAIN SVM Model |  |  |  |  |  |  |  |  |  |  |  |
|  |  | VALIDATION SVM Model |  |  |  |  |  |  |  |  |  |  |  |
| VALIDATION Ensemble Methods |  | TEST Generative Models |  |  |  |  |  |  |  |  |  |  |  |
|  |  | TRAIN Generative Models |  | -60.500 |  | 39.467 |  | 15 |  | -1.533 |  | 1.000 |  |
|  |  | VALIDATION Generative Models |  |  |  |  |  |  |  |  |  |  |  |
|  |  | TEST Regression Methods |  | -67.083 |  | 30.143 |  | 15 |  | -2.225 |  | 1.000 |  |
|  |  | TRAIN Regression Methods |  | -48.100 |  | 39.467 |  | 15 |  | -1.219 |  | 1.000 |  |
|  |  | VALIDATION Regression Methods |  |  |  |  |  |  |  |  |  |  |  |
|  |  | TEST SVM Model |  | -86.400 |  | 39.467 |  | 15 |  | -2.189 |  | 1.000 |  |
|  |  | TRAIN SVM Model |  | -75.300 |  | 39.467 |  | 15 |  | -1.908 |  | 1.000 |  |
|  |  | VALIDATION SVM Model |  |  |  |  |  |  |  |  |  |  |  |
| TEST Generative Models |  | TRAIN Generative Models |  |  |  |  |  |  |  |  |  |  |  |
|  |  | VALIDATION Generative Models |  |  |  |  |  |  |  |  |  |  |  |
|  |  | TEST Regression Methods |  |  |  |  |  |  |  |  |  |  |  |
|  |  | TRAIN Regression Methods |  |  |  |  |  |  |  |  |  |  |  |
|  |  | VALIDATION Regression Methods |  |  |  |  |  |  |  |  |  |  |  |
|  |  | TEST SVM Model |  |  |  |  |  |  |  |  |  |  |  |
|  |  | TRAIN SVM Model |  |  |  |  |  |  |  |  |  |  |  |
|  |  | VALIDATION SVM Model |  |  |  |  |  |  |  |  |  |  |  |
| TRAIN Generative Models |  | VALIDATION Generative Models |  |  |  |  |  |  |  |  |  |  |  |
|  |  | TEST Regression Methods |  | -6.583 |  | 30.143 |  | 15 |  | -0.218 |  | 1.000 |  |
|  |  | TRAIN Regression Methods |  | 12.400 |  | 39.467 |  | 15 |  | 0.314 |  | 1.000 |  |
|  |  | VALIDATION Regression Methods |  |  |  |  |  |  |  |  |  |  |  |
|  |  | TEST SVM Model |  | -25.900 |  | 39.467 |  | 15 |  | -0.656 |  | 1.000 |  |
|  |  | TRAIN SVM Model |  | -14.800 |  | 39.467 |  | 15 |  | -0.375 |  | 1.000 |  |
|  |  | VALIDATION SVM Model |  |  |  |  |  |  |  |  |  |  |  |
| VALIDATION Generative Models |  | TEST Regression Methods |  |  |  |  |  |  |  |  |  |  |  |
|  |  | TRAIN Regression Methods |  |  |  |  |  |  |  |  |  |  |  |
|  |  | VALIDATION Regression Methods |  |  |  |  |  |  |  |  |  |  |  |
|  |  | TEST SVM Model |  |  |  |  |  |  |  |  |  |  |  |
|  |  | TRAIN SVM Model |  |  |  |  |  |  |  |  |  |  |  |
|  |  | VALIDATION SVM Model |  |  |  |  |  |  |  |  |  |  |  |
| TEST Regression Methods |  | TRAIN Regression Methods |  | 18.983 |  | 30.143 |  | 15 |  | 0.630 |  | 1.000 |  |
|  |  | VALIDATION Regression Methods |  |  |  |  |  |  |  |  |  |  |  |
|  |  | TEST SVM Model |  | -19.317 |  | 30.143 |  | 15 |  | -0.641 |  | 1.000 |  |
|  |  | TRAIN SVM Model |  | -8.217 |  | 30.143 |  | 15 |  | -0.273 |  | 1.000 |  |
|  |  | VALIDATION SVM Model |  |  |  |  |  |  |  |  |  |  |  |
| TRAIN Regression Methods |  | VALIDATION Regression Methods |  |  |  |  |  |  |  |  |  |  |  |
|  |  | TEST SVM Model |  | -38.300 |  | 39.467 |  | 15 |  | -0.970 |  | 1.000 |  |
|  |  | TRAIN SVM Model |  | -27.200 |  | 39.467 |  | 15 |  | -0.689 |  | 1.000 |  |
|  |  | VALIDATION SVM Model |  |  |  |  |  |  |  |  |  |  |  |
| VALIDATION Regression Methods |  | TEST SVM Model |  |  |  |  |  |  |  |  |  |  |  |
|  |  | TRAIN SVM Model |  |  |  |  |  |  |  |  |  |  |  |
|  |  | VALIDATION SVM Model |  |  |  |  |  |  |  |  |  |  |  |
| TEST SVM Model |  | TRAIN SVM Model |  | 11.100 |  | 39.467 |  | 15 |  | 0.281 |  | 1.000 |  |
|  |  | VALIDATION SVM Model |  |  |  |  |  |  |  |  |  |  |  |
| TRAIN SVM Model |  | VALIDATION SVM Model |  |  |  |  |  |  |  |  |  |  |  |
|  | | | | | | | | | | | | | |
| *Note.*  P-value adjusted for comparing a family of 45 estimates. | | | | | | | | | | | | | |
| *Note.*  Some parameters were not estimable due to missingness. | | | | | | | | | | | | | |

**COHORT X ALGORITHM SENSITIVITY**

**Descriptives**

| *Descriptives - sensitivity* | | | | | | | | | | | | | |
| --- | --- | --- | --- | --- | --- | --- | --- | --- | --- | --- | --- | --- | --- |
| Cohort Type | | Algorithm | | N | | Mean | | SD | | SE | | Coefficient of variation | |
| TEST |  | Deep Learning |  | 3 |  | 100.000 |  | 0.000 |  | 0.000 |  | 0.000 |  |
|  |  | Ensemble Methods |  | 4 |  | 76.950 |  | 10.986 |  | 5.493 |  | 0.143 |  |
|  |  | Regression Methods |  | 5 |  | 71.600 |  | 20.085 |  | 8.982 |  | 0.281 |  |
|  |  | SVM Model |  | 1 |  | 80.000 |  |  |  |  |  |  |  |
| TRAIN |  | Deep Learning |  | 3 |  | 93.500 |  | 0.000 |  | 0.000 |  | 0.000 |  |
|  |  | Generative Models |  | 1 |  | 42.900 |  |  |  |  |  |  |  |
|  |  | Regression Methods |  | 1 |  | 54.300 |  |  |  |  |  |  |  |
|  |  | SVM Model |  | 1 |  | 35.000 |  |  |  |  |  |  |  |
| VALIDATION |  | Deep Learning |  | 2 |  | 100.000 |  | 0.000 |  | 0.000 |  | 0.000 |  |
|  | | | | | | | | | | | | | |
| *Note.*  Some combinations of factors are not observed and hence omitted (6 out of 15 combinations are unobserved). | | | | | | | | | | | | | |

**Post Hoc Tests**

**Standard**

| *Post Hoc Comparisons - Cohort Type ✻ Algorithm* | | | | | | | | | | | | | |
| --- | --- | --- | --- | --- | --- | --- | --- | --- | --- | --- | --- | --- | --- |
|  | |  | | Mean Difference | | SE | | df | | t | | p_bonf_ | |
| TEST Deep Learning |  | TRAIN Deep Learning |  | 6.500 |  | 10.477 |  | 12 |  | 0.620 |  | 1.000 |  |
|  |  | VALIDATION Deep Learning |  | -1.243×10^-14^ |  | 11.714 |  | 12 |  | -1.062×10^-15^ |  | 1.000 |  |
|  |  | TEST Ensemble Methods |  | 23.050 |  | 9.800 |  | 12 |  | 2.352 |  | 1.000 |  |
|  |  | TRAIN Ensemble Methods |  |  |  |  |  |  |  |  |  |  |  |
|  |  | VALIDATION Ensemble Methods |  |  |  |  |  |  |  |  |  |  |  |
|  |  | TEST Generative Models |  |  |  |  |  |  |  |  |  |  |  |
|  |  | TRAIN Generative Models |  | 57.100 |  | 14.817 |  | 12 |  | 3.854 |  | 0.083 |  |
|  |  | VALIDATION Generative Models |  |  |  |  |  |  |  |  |  |  |  |
|  |  | TEST Regression Methods |  | 28.400 |  | 9.371 |  | 12 |  | 3.031 |  | 0.376 |  |
|  |  | TRAIN Regression Methods |  | 45.700 |  | 14.817 |  | 12 |  | 3.084 |  | 0.341 |  |
|  |  | VALIDATION Regression Methods |  |  |  |  |  |  |  |  |  |  |  |
|  |  | TEST SVM Model |  | 20.000 |  | 14.817 |  | 12 |  | 1.350 |  | 1.000 |  |
|  |  | TRAIN SVM Model |  | 65.000 |  | 14.817 |  | 12 |  | 4.387 |  | 0.032 |  |
|  |  | VALIDATION SVM Model |  |  |  |  |  |  |  |  |  |  |  |
| TRAIN Deep Learning |  | VALIDATION Deep Learning |  | -6.500 |  | 11.714 |  | 12 |  | -0.555 |  | 1.000 |  |
|  |  | TEST Ensemble Methods |  | 16.550 |  | 9.800 |  | 12 |  | 1.689 |  | 1.000 |  |
|  |  | TRAIN Ensemble Methods |  |  |  |  |  |  |  |  |  |  |  |
|  |  | VALIDATION Ensemble Methods |  |  |  |  |  |  |  |  |  |  |  |
|  |  | TEST Generative Models |  |  |  |  |  |  |  |  |  |  |  |
|  |  | TRAIN Generative Models |  | 50.600 |  | 14.817 |  | 12 |  | 3.415 |  | 0.184 |  |
|  |  | VALIDATION Generative Models |  |  |  |  |  |  |  |  |  |  |  |
|  |  | TEST Regression Methods |  | 21.900 |  | 9.371 |  | 12 |  | 2.337 |  | 1.000 |  |
|  |  | TRAIN Regression Methods |  | 39.200 |  | 14.817 |  | 12 |  | 2.646 |  | 0.769 |  |
|  |  | VALIDATION Regression Methods |  |  |  |  |  |  |  |  |  |  |  |
|  |  | TEST SVM Model |  | 13.500 |  | 14.817 |  | 12 |  | 0.911 |  | 1.000 |  |
|  |  | TRAIN SVM Model |  | 58.500 |  | 14.817 |  | 12 |  | 3.948 |  | 0.070 |  |
|  |  | VALIDATION SVM Model |  |  |  |  |  |  |  |  |  |  |  |
| VALIDATION Deep Learning |  | TEST Ensemble Methods |  | 23.050 |  | 11.112 |  | 12 |  | 2.074 |  | 1.000 |  |
|  |  | TRAIN Ensemble Methods |  |  |  |  |  |  |  |  |  |  |  |
|  |  | VALIDATION Ensemble Methods |  |  |  |  |  |  |  |  |  |  |  |
|  |  | TEST Generative Models |  |  |  |  |  |  |  |  |  |  |  |
|  |  | TRAIN Generative Models |  | 57.100 |  | 15.715 |  | 12 |  | 3.633 |  | 0.123 |  |
|  |  | VALIDATION Generative Models |  |  |  |  |  |  |  |  |  |  |  |
|  |  | TEST Regression Methods |  | 28.400 |  | 10.736 |  | 12 |  | 2.645 |  | 0.769 |  |
|  |  | TRAIN Regression Methods |  | 45.700 |  | 15.715 |  | 12 |  | 2.908 |  | 0.473 |  |
|  |  | VALIDATION Regression Methods |  |  |  |  |  |  |  |  |  |  |  |
|  |  | TEST SVM Model |  | 20.000 |  | 15.715 |  | 12 |  | 1.273 |  | 1.000 |  |
|  |  | TRAIN SVM Model |  | 65.000 |  | 15.715 |  | 12 |  | 4.136 |  | 0.050 |  |
|  |  | VALIDATION SVM Model |  |  |  |  |  |  |  |  |  |  |  |
| TEST Ensemble Methods |  | TRAIN Ensemble Methods |  |  |  |  |  |  |  |  |  |  |  |
|  |  | VALIDATION Ensemble Methods |  |  |  |  |  |  |  |  |  |  |  |
|  |  | TEST Generative Models |  |  |  |  |  |  |  |  |  |  |  |
|  |  | TRAIN Generative Models |  | 34.050 |  | 14.346 |  | 12 |  | 2.373 |  | 1.000 |  |
|  |  | VALIDATION Generative Models |  |  |  |  |  |  |  |  |  |  |  |
|  |  | TEST Regression Methods |  | 5.350 |  | 8.608 |  | 12 |  | 0.622 |  | 1.000 |  |
|  |  | TRAIN Regression Methods |  | 22.650 |  | 14.346 |  | 12 |  | 1.579 |  | 1.000 |  |
|  |  | VALIDATION Regression Methods |  |  |  |  |  |  |  |  |  |  |  |
|  |  | TEST SVM Model |  | -3.050 |  | 14.346 |  | 12 |  | -0.213 |  | 1.000 |  |
|  |  | TRAIN SVM Model |  | 41.950 |  | 14.346 |  | 12 |  | 2.924 |  | 0.459 |  |
|  |  | VALIDATION SVM Model |  |  |  |  |  |  |  |  |  |  |  |
| TRAIN Ensemble Methods |  | VALIDATION Ensemble Methods |  |  |  |  |  |  |  |  |  |  |  |
|  |  | TEST Generative Models |  |  |  |  |  |  |  |  |  |  |  |
|  |  | TRAIN Generative Models |  |  |  |  |  |  |  |  |  |  |  |
|  |  | VALIDATION Generative Models |  |  |  |  |  |  |  |  |  |  |  |
|  |  | TEST Regression Methods |  |  |  |  |  |  |  |  |  |  |  |
|  |  | TRAIN Regression Methods |  |  |  |  |  |  |  |  |  |  |  |
|  |  | VALIDATION Regression Methods |  |  |  |  |  |  |  |  |  |  |  |
|  |  | TEST SVM Model |  |  |  |  |  |  |  |  |  |  |  |
|  |  | TRAIN SVM Model |  |  |  |  |  |  |  |  |  |  |  |
|  |  | VALIDATION SVM Model |  |  |  |  |  |  |  |  |  |  |  |
| VALIDATION Ensemble Methods |  | TEST Generative Models |  |  |  |  |  |  |  |  |  |  |  |
|  |  | TRAIN Generative Models |  |  |  |  |  |  |  |  |  |  |  |
|  |  | VALIDATION Generative Models |  |  |  |  |  |  |  |  |  |  |  |
|  |  | TEST Regression Methods |  |  |  |  |  |  |  |  |  |  |  |
|  |  | TRAIN Regression Methods |  |  |  |  |  |  |  |  |  |  |  |
|  |  | VALIDATION Regression Methods |  |  |  |  |  |  |  |  |  |  |  |
|  |  | TEST SVM Model |  |  |  |  |  |  |  |  |  |  |  |
|  |  | TRAIN SVM Model |  |  |  |  |  |  |  |  |  |  |  |
|  |  | VALIDATION SVM Model |  |  |  |  |  |  |  |  |  |  |  |
| TEST Generative Models |  | TRAIN Generative Models |  |  |  |  |  |  |  |  |  |  |  |
|  |  | VALIDATION Generative Models |  |  |  |  |  |  |  |  |  |  |  |
|  |  | TEST Regression Methods |  |  |  |  |  |  |  |  |  |  |  |
|  |  | TRAIN Regression Methods |  |  |  |  |  |  |  |  |  |  |  |
|  |  | VALIDATION Regression Methods |  |  |  |  |  |  |  |  |  |  |  |
|  |  | TEST SVM Model |  |  |  |  |  |  |  |  |  |  |  |
|  |  | TRAIN SVM Model |  |  |  |  |  |  |  |  |  |  |  |
|  |  | VALIDATION SVM Model |  |  |  |  |  |  |  |  |  |  |  |
| TRAIN Generative Models |  | VALIDATION Generative Models |  |  |  |  |  |  |  |  |  |  |  |
|  |  | TEST Regression Methods |  | -28.700 |  | 14.056 |  | 12 |  | -2.042 |  | 1.000 |  |
|  |  | TRAIN Regression Methods |  | -11.400 |  | 18.147 |  | 12 |  | -0.628 |  | 1.000 |  |
|  |  | VALIDATION Regression Methods |  |  |  |  |  |  |  |  |  |  |  |
|  |  | TEST SVM Model |  | -37.100 |  | 18.147 |  | 12 |  | -2.044 |  | 1.000 |  |
|  |  | TRAIN SVM Model |  | 7.900 |  | 18.147 |  | 12 |  | 0.435 |  | 1.000 |  |
|  |  | VALIDATION SVM Model |  |  |  |  |  |  |  |  |  |  |  |
| VALIDATION Generative Models |  | TEST Regression Methods |  |  |  |  |  |  |  |  |  |  |  |
|  |  | TRAIN Regression Methods |  |  |  |  |  |  |  |  |  |  |  |
|  |  | VALIDATION Regression Methods |  |  |  |  |  |  |  |  |  |  |  |
|  |  | TEST SVM Model |  |  |  |  |  |  |  |  |  |  |  |
|  |  | TRAIN SVM Model |  |  |  |  |  |  |  |  |  |  |  |
|  |  | VALIDATION SVM Model |  |  |  |  |  |  |  |  |  |  |  |
| TEST Regression Methods |  | TRAIN Regression Methods |  | 17.300 |  | 14.056 |  | 12 |  | 1.231 |  | 1.000 |  |
|  |  | VALIDATION Regression Methods |  |  |  |  |  |  |  |  |  |  |  |
|  |  | TEST SVM Model |  | -8.400 |  | 14.056 |  | 12 |  | -0.598 |  | 1.000 |  |
|  |  | TRAIN SVM Model |  | 36.600 |  | 14.056 |  | 12 |  | 2.604 |  | 0.830 |  |
|  |  | VALIDATION SVM Model |  |  |  |  |  |  |  |  |  |  |  |
| TRAIN Regression Methods |  | VALIDATION Regression Methods |  |  |  |  |  |  |  |  |  |  |  |
|  |  | TEST SVM Model |  | -25.700 |  | 18.147 |  | 12 |  | -1.416 |  | 1.000 |  |
|  |  | TRAIN SVM Model |  | 19.300 |  | 18.147 |  | 12 |  | 1.064 |  | 1.000 |  |
|  |  | VALIDATION SVM Model |  |  |  |  |  |  |  |  |  |  |  |
| VALIDATION Regression Methods |  | TEST SVM Model |  |  |  |  |  |  |  |  |  |  |  |
|  |  | TRAIN SVM Model |  |  |  |  |  |  |  |  |  |  |  |
|  |  | VALIDATION SVM Model |  |  |  |  |  |  |  |  |  |  |  |
| TEST SVM Model |  | TRAIN SVM Model |  | 45.000 |  | 18.147 |  | 12 |  | 2.480 |  | 1.000 |  |
|  |  | VALIDATION SVM Model |  |  |  |  |  |  |  |  |  |  |  |
| TRAIN SVM Model |  | VALIDATION SVM Model |  |  |  |  |  |  |  |  |  |  |  |
|  | | | | | | | | | | | | | |
| *Note.*  P-value adjusted for comparing a family of 36 estimates. | | | | | | | | | | | | | |
| *Note.*  Some parameters were not estimable due to missingness. | | | | | | | | | | | | | |

**COHORT X ALGORITHM SPECIFICITY**

| *Descriptives - specificity* | | | | | | | | | | | | | |
| --- | --- | --- | --- | --- | --- | --- | --- | --- | --- | --- | --- | --- | --- |
| Cohort Type | | Algorithm | | N | | Mean | | SD | | SE | | Coefficient of variation | |
| TEST |  | Deep Learning |  | 3 |  | 85.700 |  | 0.000 |  | 0.000 |  | 0.000 |  |
|  |  | Ensemble Methods |  | 4 |  | 74.225 |  | 12.935 |  | 6.467 |  | 0.174 |  |
|  |  | Regression Methods |  | 5 |  | 80.540 |  | 23.271 |  | 10.407 |  | 0.289 |  |
|  |  | SVM Model |  | 1 |  | 88.200 |  |  |  |  |  |  |  |
| TRAIN |  | Deep Learning |  | 3 |  | 91.700 |  | 0.000 |  | 0.000 |  | 0.000 |  |
|  |  | Generative Models |  | 1 |  | 73.900 |  |  |  |  |  |  |  |
|  |  | Regression Methods |  | 1 |  | 43.500 |  |  |  |  |  |  |  |
|  |  | SVM Model |  | 1 |  | 88.000 |  |  |  |  |  |  |  |
| VALIDATION |  | Deep Learning |  | 2 |  | 92.000 |  | 11.314 |  | 8.000 |  | 0.123 |  |
|  | | | | | | | | | | | | | |
| *Note.*  Some combinations of factors are not observed and hence omitted (6 out of 15 combinations are unobserved). | | | | | | | | | | | | | |

**Post Hoc Tests**

**Standard**

| *Post Hoc Comparisons - Cohort Type ✻ Algorithm* | | | | | | | | | | | | | | | | | | |
| --- | --- | --- | --- | --- | --- | --- | --- | --- | --- | --- | --- | --- | --- | --- | --- | --- | --- | --- |
|  | | | |  | | | | Mean Difference | | SE | | df | | | t | | p_bonf_ | |
| TEST Deep Learning | | |  | TRAIN Deep Learning | | |  | -6.000 |  | 12.464 |  | 12 | |  | -0.481 |  | 1.000 |  |
|  | | |  | VALIDATION Deep Learning | | |  | -6.300 |  | 13.935 |  | 12 | |  | -0.452 |  | 1.000 |  |
|  | | |  | TEST Ensemble Methods | | |  | 11.475 |  | 11.659 |  | 12 | |  | 0.984 |  | 1.000 |  |
|  | | |  | TRAIN Ensemble Methods | | |  |  |  |  |  |  | |  |  |  |  |  |
|  | | |  | VALIDATION Ensemble Methods | | |  |  |  |  |  |  | |  |  |  |  |  |
|  | | |  | TEST Generative Models | | |  |  |  |  |  |  | |  |  |  |  |  |
|  | | |  | TRAIN Generative Models | | |  | 11.800 |  | 17.626 |  | 12 | |  | 0.669 |  | 1.000 |  |
|  | | |  | VALIDATION Generative Models | | |  |  |  |  |  |  | |  |  |  |  |  |
|  | | |  | TEST Regression Methods | | |  | 5.160 |  | 11.148 |  | 12 | |  | 0.463 |  | 1.000 |  |
|  | | |  | TRAIN Regression Methods | | |  | 42.200 |  | 17.626 |  | 12 | |  | 2.394 |  | 1.000 |  |
|  | | |  | VALIDATION Regression Methods | | |  |  |  |  |  |  | |  |  |  |  |  |
|  | | |  | TEST SVM Model | | |  | -2.500 |  | 17.626 |  | 12 | |  | -0.142 |  | 1.000 |  |
|  | | |  | TRAIN SVM Model | | |  | -2.300 |  | 17.626 |  | 12 | |  | -0.130 |  | 1.000 |  |
|  | | |  | VALIDATION SVM Model | | |  |  |  |  |  |  | |  |  |  |  |  |
| TRAIN Deep Learning | | |  | VALIDATION Deep Learning | | |  | -0.300 |  | 13.935 |  | 12 | |  | -0.022 |  | 1.000 |  |
|  | | |  | TEST Ensemble Methods | | |  | 17.475 |  | 11.659 |  | 12 | |  | 1.499 |  | 1.000 |  |
|  | | |  | TRAIN Ensemble Methods | | |  |  |  |  |  |  | |  |  |  |  |  |
|  | | |  | VALIDATION Ensemble Methods | | |  |  |  |  |  |  | |  |  |  |  |  |
|  | | |  | TEST Generative Models | | |  |  |  |  |  |  | |  |  |  |  |  |
|  | | |  | TRAIN Generative Models | | |  | 17.800 |  | 17.626 |  | 12 | |  | 1.010 |  | 1.000 |  |
|  | | |  | VALIDATION Generative Models | | |  |  |  |  |  |  | |  |  |  |  |  |
|  | | |  | TEST Regression Methods | | |  | 11.160 |  | 11.148 |  | 12 | |  | 1.001 |  | 1.000 |  |
|  | | |  | TRAIN Regression Methods | | |  | 48.200 |  | 17.626 |  | 12 | |  | 2.735 |  | 0.652 |  |
|  | | |  | VALIDATION Regression Methods | | |  |  |  |  |  |  | |  |  |  |  |  |
|  | | |  | TEST SVM Model | | |  | 3.500 |  | 17.626 |  | 12 | |  | 0.199 |  | 1.000 |  |
|  | | |  | TRAIN SVM Model | | |  | 3.700 |  | 17.626 |  | 12 | |  | 0.210 |  | 1.000 |  |
|  | | |  | VALIDATION SVM Model | | |  |  |  |  |  |  | |  |  |  |  |  |
| VALIDATION Deep Learning | | |  | TEST Ensemble Methods | | |  | 17.775 |  | 13.220 |  | 12 | |  | 1.345 |  | 1.000 |  |
|  | | |  | TRAIN Ensemble Methods | | |  |  |  |  |  |  | |  |  |  |  |  |
|  | | |  | VALIDATION Ensemble Methods | | |  |  |  |  |  |  | |  |  |  |  |  |
|  | | |  | TEST Generative Models | | |  |  |  |  |  |  | |  |  |  |  |  |
|  | | |  | TRAIN Generative Models | | |  | 18.100 |  | 18.695 |  | 12 | |  | 0.968 |  | 1.000 |  |
|  | | |  | VALIDATION Generative Models | | |  |  |  |  |  |  | |  |  |  |  |  |
|  | | |  | TEST Regression Methods | | |  | 11.460 |  | 12.771 |  | 12 | |  | 0.897 |  | 1.000 |  |
|  | | |  | TRAIN Regression Methods | | |  | 48.500 |  | 18.695 |  | 12 | |  | 2.594 |  | 0.845 |  |
|  | | |  | VALIDATION Regression Methods | | |  |  |  |  |  |  | |  |  |  |  |  |
|  | | |  | TEST SVM Model | | |  | 3.800 |  | 18.695 |  | 12 | |  | 0.203 |  | 1.000 |  |
|  | | |  | TRAIN SVM Model | | |  | 4.000 |  | 18.695 |  | 12 | |  | 0.214 |  | 1.000 |  |
|  | | |  | VALIDATION SVM Model | | |  |  |  |  |  |  | |  |  |  |  |  |
| TEST Ensemble Methods | | |  | TRAIN Ensemble Methods | | |  |  |  |  |  |  | |  |  |  |  |  |
|  | | |  | VALIDATION Ensemble Methods | | |  |  |  |  |  |  | |  |  |  |  |  |
|  | | |  | TEST Generative Models | | |  |  |  |  |  |  | |  |  |  |  |  |
|  | | |  | TRAIN Generative Models | | |  | 0.325 |  | 17.067 |  | 12 | |  | 0.019 |  | 1.000 |  |
|  | | |  | VALIDATION Generative Models | | |  |  |  |  |  |  | |  |  |  |  |  |
|  | | |  | TEST Regression Methods | | |  | -6.315 |  | 10.240 |  | 12 | |  | -0.617 |  | 1.000 |  |
|  | | |  | TRAIN Regression Methods | | |  | 30.725 |  | 17.067 |  | 12 | |  | 1.800 |  | 1.000 |  |
|  | | |  | VALIDATION Regression Methods | | |  |  |  |  |  |  | |  |  |  |  |  |
|  | | |  | TEST SVM Model | | |  | -13.975 |  | 17.067 |  | 12 | |  | -0.819 |  | 1.000 |  |
|  | | |  | TRAIN SVM Model | | |  | -13.775 |  | 17.067 |  | 12 | |  | -0.807 |  | 1.000 |  |
|  | | |  | VALIDATION SVM Model | | |  |  |  |  |  |  | |  |  |  |  |  |
| TRAIN Ensemble Methods | | |  | VALIDATION Ensemble Methods | | |  |  |  |  |  |  | |  |  |  |  |  |
|  | | |  | TEST Generative Models | | |  |  |  |  |  |  | |  |  |  |  |  |
|  | | |  | TRAIN Generative Models | | |  |  |  |  |  |  | |  |  |  |  |  |
|  | | |  | VALIDATION Generative Models | | |  |  |  |  |  |  | |  |  |  |  |  |
|  | | |  | TEST Regression Methods | | |  |  |  |  |  |  | |  |  |  |  |  |
|  | | |  | TRAIN Regression Methods | | |  |  |  |  |  |  | |  |  |  |  |  |
|  | | |  | VALIDATION Regression Methods | | |  |  |  |  |  |  | |  |  |  |  |  |
|  | | |  | TEST SVM Model | | |  |  |  |  |  |  | |  |  |  |  |  |
|  | | |  | TRAIN SVM Model | | |  |  |  |  |  |  | |  |  |  |  |  |
|  | | |  | VALIDATION SVM Model | | |  |  |  |  |  |  | |  |  |  |  |  |
| VALIDATION Ensemble Methods | | |  | TEST Generative Models | | |  |  |  |  |  |  | |  |  |  |  |  |
|  | | |  | TRAIN Generative Models | | |  |  |  |  |  |  | |  |  |  |  |  |
|  | | |  | VALIDATION Generative Models | | |  |  |  |  |  |  | |  |  |  |  |  |
|  | | |  | TEST Regression Methods | | |  |  |  |  |  |  | |  |  |  |  |  |
|  | | |  | TRAIN Regression Methods | | |  |  |  |  |  |  | |  |  |  |  |  |
|  | | |  | VALIDATION Regression Methods | | |  |  |  |  |  |  | |  |  |  |  |  |
|  | | |  | TEST SVM Model | | |  |  |  |  |  |  | |  |  |  |  |  |
|  | | |  | TRAIN SVM Model | | |  |  |  |  |  |  | |  |  |  |  |  |
|  | | |  | VALIDATION SVM Model | | |  |  |  |  |  |  | |  |  |  |  |  |
| TEST Generative Models | | |  | TRAIN Generative Models | | |  |  |  |  |  |  | |  |  |  |  |  |
|  | | |  | VALIDATION Generative Models | | |  |  |  |  |  |  | |  |  |  |  |  |
|  | | |  | TEST Regression Methods | | |  |  |  |  |  |  | |  |  |  |  |  |
|  | | |  | TRAIN Regression Methods | | |  |  |  |  |  |  | |  |  |  |  |  |
|  | | |  | VALIDATION Regression Methods | | |  |  |  |  |  |  | |  |  |  |  |  |
|  | | |  | TEST SVM Model | | |  |  |  |  |  |  | |  |  |  |  |  |
|  | | |  | TRAIN SVM Model | | |  |  |  |  |  |  | |  |  |  |  |  |
|  | | |  | VALIDATION SVM Model | | |  |  |  |  |  |  | |  |  |  |  |  |
| TRAIN Generative Models | | |  | VALIDATION Generative Models | | |  |  |  |  |  |  | |  |  |  |  |  |
|  | | |  | TEST Regression Methods | | |  | -6.640 |  | 16.722 |  | 12 | |  | -0.397 |  | 1.000 |  |
|  | | |  | TRAIN Regression Methods | | |  | 30.400 |  | 21.588 |  | 12 | |  | 1.408 |  | 1.000 |  |
|  | | |  | VALIDATION Regression Methods | | |  |  |  |  |  |  | |  |  |  |  |  |
|  | | |  | TEST SVM Model | | |  | -14.300 |  | 21.588 |  | 12 | |  | -0.662 |  | 1.000 |  |
|  | | |  | TRAIN SVM Model | | |  | -14.100 |  | 21.588 |  | 12 | |  | -0.653 |  | 1.000 |  |
|  | | |  | VALIDATION SVM Model | | |  |  |  |  |  |  | |  |  |  |  |  |
| VALIDATION Generative Models | | |  | TEST Regression Methods | | |  |  |  |  |  |  | |  |  |  |  |  |
|  | | |  | TRAIN Regression Methods | | |  |  |  |  |  |  | |  |  |  |  |  |
|  | | |  | VALIDATION Regression Methods | | |  |  |  |  |  |  | |  |  |  |  |  |
|  | | |  | TEST SVM Model | | |  |  |  |  |  |  | |  |  |  |  |  |
|  | | |  | TRAIN SVM Model | | |  |  |  |  |  |  | |  |  |  |  |  |
|  | | |  | VALIDATION SVM Model | | |  |  |  |  |  |  | |  |  |  |  |  |
| TEST Regression Methods | | |  | TRAIN Regression Methods | | |  | 37.040 |  | 16.722 |  | 12 | |  | 2.215 |  | 1.000 |  |
|  | | |  | VALIDATION Regression Methods | | |  |  |  |  |  |  | |  |  |  |  |  |
|  | | |  | TEST SVM Model | | |  | -7.660 |  | 16.722 |  | 12 | |  | -0.458 |  | 1.000 |  |
|  | | |  | TRAIN SVM Model | | |  | -7.460 |  | 16.722 |  | 12 | |  | -0.446 |  | 1.000 |  |
|  | | |  | VALIDATION SVM Model | | |  |  |  |  |  |  | |  |  |  |  |  |
| TRAIN Regression Methods | | |  | VALIDATION Regression Methods | | |  |  |  |  |  |  | |  |  |  |  |  |
|  | | |  | TEST SVM Model | | |  | -44.700 |  | 21.588 |  | 12 | |  | -2.071 |  | 1.000 |  |
|  | | |  | TRAIN SVM Model | | |  | -44.500 |  | 21.588 |  | 12 | |  | -2.061 |  | 1.000 |  |
|  | | |  | VALIDATION SVM Model | | |  |  |  |  |  |  | |  |  |  |  |  |
| VALIDATION Regression Methods | | |  | TEST SVM Model | | |  |  |  |  |  |  | |  |  |  |  |  |
|  | | |  | TRAIN SVM Model | | |  |  |  |  |  |  | |  |  |  |  |  |
|  | | |  | VALIDATION SVM Model | | |  |  |  |  |  |  | |  |  |  |  |  |
| TEST SVM Model | | |  | TRAIN SVM Model | | |  | 0.200 |  | 21.588 |  | 12 | |  | 0.009 |  | 1.000 |  |
|  | | |  | VALIDATION SVM Model | | |  |  |  |  |  |  | |  |  |  |  |  |
| TRAIN SVM Model | | |  | VALIDATION SVM Model | | |  |  |  |  |  |  | |  |  |  |  |  |
|  | | | | | | | | | | | | | | | | | | |
| *Note.*  P-value adjusted for comparing a family of 36 estimates. | | | | | | | | | | | | | | | | | | |
| *Note.*  Some parameters were not estimable due to missingness. | | | | | | | | | | | | | | | | | | |
| *ANOVA - AUC-ROC*  *Your design contains empty cells. Please try a different type of sum of squares.* | | | | | | | | | | | | | | | | | | |
| Cases | | Sum of Squares | | df | | Mean Square | | F | | p | | |  |  |  |  |  |  |
|  |  |  |  |  |  |  |  |  |  |  |  | |  |  |  |  |  |  |
|  |  |  |  |  |  |  |  |  |  |  |  | |  |  |  |  |  |  |
|  |  |  |  |  |  |  |  |  |  |  |  | |  |  |  |  |  |  |
|  | | | | | | | | | | | | |  |  |  |  |  |  |
| *Note.*  Type III Sum of Squares | | | | | | | | | | | | |  |  |  |  |  |  |

**COHORT X ALGORITHM AUC-ROC**

| *Descriptives - AUC-ROC* | | | | | | | | | | | | | |
| --- | --- | --- | --- | --- | --- | --- | --- | --- | --- | --- | --- | --- | --- |
| Cohort Type | | Algorithm | | N | | Mean | | SD | | SE | | Coefficient of variation | |
| TEST |  | Deep Learning |  | 2 |  | 0.960 |  | 0.000 |  | 0.000 |  | 0.000 |  |
|  |  | Ensemble Methods |  | 5 |  | 18.249 |  | 38.992 |  | 17.438 |  | 2.137 |  |
|  |  | Regression Methods |  | 4 |  | 0.800 |  | 0.158 |  | 0.079 |  | 0.198 |  |
| TRAIN |  |  |  | 1 |  | 0.459 |  |  |  |  |  |  |  |
| VALIDATION |  | Deep Learning |  | 1 |  | 1.000 |  |  |  |  |  |  |  |
|  |  | Ensemble Methods |  | 1 |  | 0.880 |  |  |  |  |  |  |  |
|  | | | | | | | | | | | | | |
| *Note.*  Some combinations of factors are not observed and hence omitted (3 out of 9 combinations are unobserved). | | | | | | | | | | | | | |

**Post Hoc Tests**

**Standard**

| *Post Hoc Comparisons - Algorithm* | | | | | | | | | | | | | |
| --- | --- | --- | --- | --- | --- | --- | --- | --- | --- | --- | --- | --- | --- |
|  | |  | | Mean Difference | | SE | | df | | t | | p_bonf_ | |
| Deep Learning |  | Ensemble Methods |  |  |  |  |  |  |  |  |  |  |  |
|  |  | Regression Methods |  |  |  |  |  |  |  |  |  |  |  |
| Ensemble Methods |  | Regression Methods |  |  |  |  |  |  |  |  |  |  |  |
|  | | | | | | | | | | | | | |
| *Note.*  P-value adjusted for comparing a family of 1 estimates. | | | | | | | | | | | | | |
| *Note.*  Results are averaged over the levels of: Cohort Type | | | | | | | | | | | | | |
| *Note.*  Some parameters were not estimable due to missingness. | | | | | | | | | | | | | |

**Marginal Means**

| *Marginal Means - Algorithm* | | | | | | | | | |
| --- | --- | --- | --- | --- | --- | --- | --- | --- | --- |
|  | | | | 95% CI for Mean Difference | | | |  | |
| Algorithm | | Marginal Mean | | Lower | | Upper | | SE | |
| Deep Learning |  |  |  |  |  |  |  |  |  |
| Ensemble Methods |  |  |  |  |  |  |  |  |  |
| Regression Methods |  |  |  |  |  |  |  |  |  |
|  | | | | | | | | | |

**COHORT X ALGORITHM PPV**

| *Descriptives - PPV* | | | | | | | | | | | | | |
| --- | --- | --- | --- | --- | --- | --- | --- | --- | --- | --- | --- | --- | --- |
| Cohort Type | | Algorithm | | N | | Mean | | SD | | SE | | Coefficient of variation | |
| TEST |  | Deep Learning |  | 4 |  | 57.275 |  | 38.052 |  | 19.026 |  | 0.664 |  |
|  |  | Ensemble Methods |  | 4 |  | 0.685 |  | 0.266 |  | 0.133 |  | 0.388 |  |
|  |  | Regression Methods |  | 5 |  | 0.682 |  | 0.314 |  | 0.140 |  | 0.460 |  |
|  |  | SVM Model |  | 1 |  | 66.700 |  |  |  |  |  |  |  |
| TRAIN |  | Deep Learning |  | 3 |  | 85.000 |  | 0.000 |  | 0.000 |  | 0.000 |  |
|  |  | Generative Models |  | 1 |  | 0.560 |  |  |  |  |  |  |  |
|  |  | Regression Methods |  | 1 |  | 0.420 |  |  |  |  |  |  |  |
|  |  | SVM Model |  | 1 |  | 56.200 |  |  |  |  |  |  |  |
| VALIDATION |  | Deep Learning |  | 2 |  | 0.835 |  | 0.233 |  | 0.165 |  | 0.279 |  |
|  | | | | | | | | | | | | | |
| *Note.*  Some combinations of factors are not observed and hence omitted (6 out of 15 combinations are unobserved). | | | | | | | | | | | | | |

**Post Hoc Tests**

**Standard**

| *Post Hoc Comparisons - Cohort Type ✻ Algorithm* | | | | | | | | | | | | | |
| --- | --- | --- | --- | --- | --- | --- | --- | --- | --- | --- | --- | --- | --- |
|  | |  | | Mean Difference | | SE | | df | | t | | p_bonf_ | |
| TEST Deep Learning |  | TRAIN Deep Learning |  | -27.725 |  | 13.962 |  | 13 |  | -1.986 |  | 1.000 |  |
|  |  | VALIDATION Deep Learning |  | 56.440 |  | 15.832 |  | 13 |  | 3.565 |  | 0.124 |  |
|  |  | TEST Ensemble Methods |  | 56.590 |  | 12.927 |  | 13 |  | 4.378 |  | 0.027 |  |
|  |  | TRAIN Ensemble Methods |  |  |  |  |  |  |  |  |  |  |  |
|  |  | VALIDATION Ensemble Methods |  |  |  |  |  |  |  |  |  |  |  |
|  |  | TEST Generative Models |  |  |  |  |  |  |  |  |  |  |  |
|  |  | TRAIN Generative Models |  | 56.715 |  | 20.439 |  | 13 |  | 2.775 |  | 0.568 |  |
|  |  | VALIDATION Generative Models |  |  |  |  |  |  |  |  |  |  |  |
|  |  | TEST Regression Methods |  | 56.593 |  | 12.263 |  | 13 |  | 4.615 |  | 0.017 |  |
|  |  | TRAIN Regression Methods |  | 56.855 |  | 20.439 |  | 13 |  | 2.782 |  | 0.560 |  |
|  |  | VALIDATION Regression Methods |  |  |  |  |  |  |  |  |  |  |  |
|  |  | TEST SVM Model |  | -9.425 |  | 20.439 |  | 13 |  | -0.461 |  | 1.000 |  |
|  |  | TRAIN SVM Model |  | 1.075 |  | 20.439 |  | 13 |  | 0.053 |  | 1.000 |  |
|  |  | VALIDATION SVM Model |  |  |  |  |  |  |  |  |  |  |  |
| TRAIN Deep Learning |  | VALIDATION Deep Learning |  | 84.165 |  | 16.688 |  | 13 |  | 5.043 |  | 0.008 |  |
|  |  | TEST Ensemble Methods |  | 84.315 |  | 13.962 |  | 13 |  | 6.039 |  | 0.002 |  |
|  |  | TRAIN Ensemble Methods |  |  |  |  |  |  |  |  |  |  |  |
|  |  | VALIDATION Ensemble Methods |  |  |  |  |  |  |  |  |  |  |  |
|  |  | TEST Generative Models |  |  |  |  |  |  |  |  |  |  |  |
|  |  | TRAIN Generative Models |  | 84.440 |  | 21.109 |  | 13 |  | 4.000 |  | 0.054 |  |
|  |  | VALIDATION Generative Models |  |  |  |  |  |  |  |  |  |  |  |
|  |  | TEST Regression Methods |  | 84.318 |  | 13.350 |  | 13 |  | 6.316 |  | < .001 |  |
|  |  | TRAIN Regression Methods |  | 84.580 |  | 21.109 |  | 13 |  | 4.007 |  | 0.054 |  |
|  |  | VALIDATION Regression Methods |  |  |  |  |  |  |  |  |  |  |  |
|  |  | TEST SVM Model |  | 18.300 |  | 21.109 |  | 13 |  | 0.867 |  | 1.000 |  |
|  |  | TRAIN SVM Model |  | 28.800 |  | 21.109 |  | 13 |  | 1.364 |  | 1.000 |  |
|  |  | VALIDATION SVM Model |  |  |  |  |  |  |  |  |  |  |  |
| VALIDATION Deep Learning |  | TEST Ensemble Methods |  | 0.150 |  | 15.832 |  | 13 |  | 0.009 |  | 1.000 |  |
|  |  | TRAIN Ensemble Methods |  |  |  |  |  |  |  |  |  |  |  |
|  |  | VALIDATION Ensemble Methods |  |  |  |  |  |  |  |  |  |  |  |
|  |  | TEST Generative Models |  |  |  |  |  |  |  |  |  |  |  |
|  |  | TRAIN Generative Models |  | 0.275 |  | 22.389 |  | 13 |  | 0.012 |  | 1.000 |  |
|  |  | VALIDATION Generative Models |  |  |  |  |  |  |  |  |  |  |  |
|  |  | TEST Regression Methods |  | 0.153 |  | 15.295 |  | 13 |  | 0.010 |  | 1.000 |  |
|  |  | TRAIN Regression Methods |  | 0.415 |  | 22.389 |  | 13 |  | 0.019 |  | 1.000 |  |
|  |  | VALIDATION Regression Methods |  |  |  |  |  |  |  |  |  |  |  |
|  |  | TEST SVM Model |  | -65.865 |  | 22.389 |  | 13 |  | -2.942 |  | 0.412 |  |
|  |  | TRAIN SVM Model |  | -55.365 |  | 22.389 |  | 13 |  | -2.473 |  | 1.000 |  |
|  |  | VALIDATION SVM Model |  |  |  |  |  |  |  |  |  |  |  |
| TEST Ensemble Methods |  | TRAIN Ensemble Methods |  |  |  |  |  |  |  |  |  |  |  |
|  |  | VALIDATION Ensemble Methods |  |  |  |  |  |  |  |  |  |  |  |
|  |  | TEST Generative Models |  |  |  |  |  |  |  |  |  |  |  |
|  |  | TRAIN Generative Models |  | 0.125 |  | 20.439 |  | 13 |  | 0.006 |  | 1.000 |  |
|  |  | VALIDATION Generative Models |  |  |  |  |  |  |  |  |  |  |  |
|  |  | TEST Regression Methods |  | 0.003 |  | 12.263 |  | 13 |  | 2.446×10^-4^ |  | 1.000 |  |
|  |  | TRAIN Regression Methods |  | 0.265 |  | 20.439 |  | 13 |  | 0.013 |  | 1.000 |  |
|  |  | VALIDATION Regression Methods |  |  |  |  |  |  |  |  |  |  |  |
|  |  | TEST SVM Model |  | -66.015 |  | 20.439 |  | 13 |  | -3.230 |  | 0.237 |  |
|  |  | TRAIN SVM Model |  | -55.515 |  | 20.439 |  | 13 |  | -2.716 |  | 0.635 |  |
|  |  | VALIDATION SVM Model |  |  |  |  |  |  |  |  |  |  |  |
| TRAIN Ensemble Methods |  | VALIDATION Ensemble Methods |  |  |  |  |  |  |  |  |  |  |  |
|  |  | TEST Generative Models |  |  |  |  |  |  |  |  |  |  |  |
|  |  | TRAIN Generative Models |  |  |  |  |  |  |  |  |  |  |  |
|  |  | VALIDATION Generative Models |  |  |  |  |  |  |  |  |  |  |  |
|  |  | TEST Regression Methods |  |  |  |  |  |  |  |  |  |  |  |
|  |  | TRAIN Regression Methods |  |  |  |  |  |  |  |  |  |  |  |
|  |  | VALIDATION Regression Methods |  |  |  |  |  |  |  |  |  |  |  |
|  |  | TEST SVM Model |  |  |  |  |  |  |  |  |  |  |  |
|  |  | TRAIN SVM Model |  |  |  |  |  |  |  |  |  |  |  |
|  |  | VALIDATION SVM Model |  |  |  |  |  |  |  |  |  |  |  |
| VALIDATION Ensemble Methods |  | TEST Generative Models |  |  |  |  |  |  |  |  |  |  |  |
|  |  | TRAIN Generative Models |  |  |  |  |  |  |  |  |  |  |  |
|  |  | VALIDATION Generative Models |  |  |  |  |  |  |  |  |  |  |  |
|  |  | TEST Regression Methods |  |  |  |  |  |  |  |  |  |  |  |
|  |  | TRAIN Regression Methods |  |  |  |  |  |  |  |  |  |  |  |
|  |  | VALIDATION Regression Methods |  |  |  |  |  |  |  |  |  |  |  |
|  |  | TEST SVM Model |  |  |  |  |  |  |  |  |  |  |  |
|  |  | TRAIN SVM Model |  |  |  |  |  |  |  |  |  |  |  |
|  |  | VALIDATION SVM Model |  |  |  |  |  |  |  |  |  |  |  |
| TEST Generative Models |  | TRAIN Generative Models |  |  |  |  |  |  |  |  |  |  |  |
|  |  | VALIDATION Generative Models |  |  |  |  |  |  |  |  |  |  |  |
|  |  | TEST Regression Methods |  |  |  |  |  |  |  |  |  |  |  |
|  |  | TRAIN Regression Methods |  |  |  |  |  |  |  |  |  |  |  |
|  |  | VALIDATION Regression Methods |  |  |  |  |  |  |  |  |  |  |  |
|  |  | TEST SVM Model |  |  |  |  |  |  |  |  |  |  |  |
|  |  | TRAIN SVM Model |  |  |  |  |  |  |  |  |  |  |  |
|  |  | VALIDATION SVM Model |  |  |  |  |  |  |  |  |  |  |  |
| TRAIN Generative Models |  | VALIDATION Generative Models |  |  |  |  |  |  |  |  |  |  |  |
|  |  | TEST Regression Methods |  | -0.122 |  | 20.026 |  | 13 |  | -0.006 |  | 1.000 |  |
|  |  | TRAIN Regression Methods |  | 0.140 |  | 25.853 |  | 13 |  | 0.005 |  | 1.000 |  |
|  |  | VALIDATION Regression Methods |  |  |  |  |  |  |  |  |  |  |  |
|  |  | TEST SVM Model |  | -66.140 |  | 25.853 |  | 13 |  | -2.558 |  | 0.857 |  |
|  |  | TRAIN SVM Model |  | -55.640 |  | 25.853 |  | 13 |  | -2.152 |  | 1.000 |  |
|  |  | VALIDATION SVM Model |  |  |  |  |  |  |  |  |  |  |  |
| VALIDATION Generative Models |  | TEST Regression Methods |  |  |  |  |  |  |  |  |  |  |  |
|  |  | TRAIN Regression Methods |  |  |  |  |  |  |  |  |  |  |  |
|  |  | VALIDATION Regression Methods |  |  |  |  |  |  |  |  |  |  |  |
|  |  | TEST SVM Model |  |  |  |  |  |  |  |  |  |  |  |
|  |  | TRAIN SVM Model |  |  |  |  |  |  |  |  |  |  |  |
|  |  | VALIDATION SVM Model |  |  |  |  |  |  |  |  |  |  |  |
| TEST Regression Methods |  | TRAIN Regression Methods |  | 0.262 |  | 20.026 |  | 13 |  | 0.013 |  | 1.000 |  |
|  |  | VALIDATION Regression Methods |  |  |  |  |  |  |  |  |  |  |  |
|  |  | TEST SVM Model |  | -66.018 |  | 20.026 |  | 13 |  | -3.297 |  | 0.208 |  |
|  |  | TRAIN SVM Model |  | -55.518 |  | 20.026 |  | 13 |  | -2.772 |  | 0.570 |  |
|  |  | VALIDATION SVM Model |  |  |  |  |  |  |  |  |  |  |  |
| TRAIN Regression Methods |  | VALIDATION Regression Methods |  |  |  |  |  |  |  |  |  |  |  |
|  |  | TEST SVM Model |  | -66.280 |  | 25.853 |  | 13 |  | -2.564 |  | 0.849 |  |
|  |  | TRAIN SVM Model |  | -55.780 |  | 25.853 |  | 13 |  | -2.158 |  | 1.000 |  |
|  |  | VALIDATION SVM Model |  |  |  |  |  |  |  |  |  |  |  |
| VALIDATION Regression Methods |  | TEST SVM Model |  |  |  |  |  |  |  |  |  |  |  |
|  |  | TRAIN SVM Model |  |  |  |  |  |  |  |  |  |  |  |
|  |  | VALIDATION SVM Model |  |  |  |  |  |  |  |  |  |  |  |
| TEST SVM Model |  | TRAIN SVM Model |  | 10.500 |  | 25.853 |  | 13 |  | 0.406 |  | 1.000 |  |
|  |  | VALIDATION SVM Model |  |  |  |  |  |  |  |  |  |  |  |
| TRAIN SVM Model |  | VALIDATION SVM Model |  |  |  |  |  |  |  |  |  |  |  |
|  | | | | | | | | | | | | | |
| *Note.*  P-value adjusted for comparing a family of 36 estimates. | | | | | | | | | | | | | |
| *Note.*  Some parameters were not estimable due to missingness. | | | | | | | | | | | | | |

**COHORT X ALGORITHM NPV**

| *Descriptives - NPV* | | | | | | | | | | | | | |
| --- | --- | --- | --- | --- | --- | --- | --- | --- | --- | --- | --- | --- | --- |
| Cohort Type | | Algorithm | | N | | Mean | | SD | | SE | | Coefficient of variation | |
| TEST |  | Deep Learning |  | 3 |  | 67.000 |  | 57.158 |  | 33.000 |  | 0.853 |  |
|  |  | Ensemble Methods |  | 4 |  | 0.735 |  | 0.285 |  | 0.143 |  | 0.388 |  |
|  |  | Regression Methods |  | 5 |  | 0.786 |  | 0.172 |  | 0.077 |  | 0.219 |  |
|  |  | SVM Model |  | 1 |  | 93.800 |  |  |  |  |  |  |  |
| TRAIN |  | Deep Learning |  | 3 |  | 96.000 |  | 0.000 |  | 0.000 |  | 0.000 |  |
|  |  | Generative Models |  | 1 |  | 0.630 |  |  |  |  |  |  |  |
|  |  | Regression Methods |  | 1 |  | 0.560 |  |  |  |  |  |  |  |
|  |  | SVM Model |  | 1 |  | 76.100 |  |  |  |  |  |  |  |
| VALIDATION |  | Deep Learning |  | 2 |  | 1.000 |  | 0.000 |  | 0.000 |  | 0.000 |  |
|  | | | | | | | | | | | | | |
| *Note.*  Some combinations of factors are not observed and hence omitted (6 out of 15 combinations are unobserved). | | | | | | | | | | | | | |

**Post Hoc Tests**

**Standard**

| *Post Hoc Comparisons - Cohort Type ✻ Algorithm* | | | | | | | | | | | | | |
| --- | --- | --- | --- | --- | --- | --- | --- | --- | --- | --- | --- | --- | --- |
|  | |  | | Mean Difference | | SE | | df | | t | | p_bonf_ | |
| TEST Deep Learning |  | TRAIN Deep Learning |  | -29.000 |  | 19.053 |  | 12 |  | -1.522 |  | 1.000 |  |
|  |  | VALIDATION Deep Learning |  | 66.000 |  | 21.302 |  | 12 |  | 3.098 |  | 0.332 |  |
|  |  | TEST Ensemble Methods |  | 66.265 |  | 17.823 |  | 12 |  | 3.718 |  | 0.106 |  |
|  |  | TRAIN Ensemble Methods |  |  |  |  |  |  |  |  |  |  |  |
|  |  | VALIDATION Ensemble Methods |  |  |  |  |  |  |  |  |  |  |  |
|  |  | TEST Generative Models |  |  |  |  |  |  |  |  |  |  |  |
|  |  | TRAIN Generative Models |  | 66.370 |  | 26.945 |  | 12 |  | 2.463 |  | 1.000 |  |
|  |  | VALIDATION Generative Models |  |  |  |  |  |  |  |  |  |  |  |
|  |  | TEST Regression Methods |  | 66.214 |  | 17.042 |  | 12 |  | 3.885 |  | 0.078 |  |
|  |  | TRAIN Regression Methods |  | 66.440 |  | 26.945 |  | 12 |  | 2.466 |  | 1.000 |  |
|  |  | VALIDATION Regression Methods |  |  |  |  |  |  |  |  |  |  |  |
|  |  | TEST SVM Model |  | -26.800 |  | 26.945 |  | 12 |  | -0.995 |  | 1.000 |  |
|  |  | TRAIN SVM Model |  | -9.100 |  | 26.945 |  | 12 |  | -0.338 |  | 1.000 |  |
|  |  | VALIDATION SVM Model |  |  |  |  |  |  |  |  |  |  |  |
| TRAIN Deep Learning |  | VALIDATION Deep Learning |  | 95.000 |  | 21.302 |  | 12 |  | 4.460 |  | 0.028 |  |
|  |  | TEST Ensemble Methods |  | 95.265 |  | 17.823 |  | 12 |  | 5.345 |  | 0.006 |  |
|  |  | TRAIN Ensemble Methods |  |  |  |  |  |  |  |  |  |  |  |
|  |  | VALIDATION Ensemble Methods |  |  |  |  |  |  |  |  |  |  |  |
|  |  | TEST Generative Models |  |  |  |  |  |  |  |  |  |  |  |
|  |  | TRAIN Generative Models |  | 95.370 |  | 26.945 |  | 12 |  | 3.539 |  | 0.147 |  |
|  |  | VALIDATION Generative Models |  |  |  |  |  |  |  |  |  |  |  |
|  |  | TEST Regression Methods |  | 95.214 |  | 17.042 |  | 12 |  | 5.587 |  | 0.004 |  |
|  |  | TRAIN Regression Methods |  | 95.440 |  | 26.945 |  | 12 |  | 3.542 |  | 0.146 |  |
|  |  | VALIDATION Regression Methods |  |  |  |  |  |  |  |  |  |  |  |
|  |  | TEST SVM Model |  | 2.200 |  | 26.945 |  | 12 |  | 0.082 |  | 1.000 |  |
|  |  | TRAIN SVM Model |  | 19.900 |  | 26.945 |  | 12 |  | 0.739 |  | 1.000 |  |
|  |  | VALIDATION SVM Model |  |  |  |  |  |  |  |  |  |  |  |
| VALIDATION Deep Learning |  | TEST Ensemble Methods |  | 0.265 |  | 20.209 |  | 12 |  | 0.013 |  | 1.000 |  |
|  |  | TRAIN Ensemble Methods |  |  |  |  |  |  |  |  |  |  |  |
|  |  | VALIDATION Ensemble Methods |  |  |  |  |  |  |  |  |  |  |  |
|  |  | TEST Generative Models |  |  |  |  |  |  |  |  |  |  |  |
|  |  | TRAIN Generative Models |  | 0.370 |  | 28.580 |  | 12 |  | 0.013 |  | 1.000 |  |
|  |  | VALIDATION Generative Models |  |  |  |  |  |  |  |  |  |  |  |
|  |  | TEST Regression Methods |  | 0.214 |  | 19.524 |  | 12 |  | 0.011 |  | 1.000 |  |
|  |  | TRAIN Regression Methods |  | 0.440 |  | 28.580 |  | 12 |  | 0.015 |  | 1.000 |  |
|  |  | VALIDATION Regression Methods |  |  |  |  |  |  |  |  |  |  |  |
|  |  | TEST SVM Model |  | -92.800 |  | 28.580 |  | 12 |  | -3.247 |  | 0.252 |  |
|  |  | TRAIN SVM Model |  | -75.100 |  | 28.580 |  | 12 |  | -2.628 |  | 0.794 |  |
|  |  | VALIDATION SVM Model |  |  |  |  |  |  |  |  |  |  |  |
| TEST Ensemble Methods |  | TRAIN Ensemble Methods |  |  |  |  |  |  |  |  |  |  |  |
|  |  | VALIDATION Ensemble Methods |  |  |  |  |  |  |  |  |  |  |  |
|  |  | TEST Generative Models |  |  |  |  |  |  |  |  |  |  |  |
|  |  | TRAIN Generative Models |  | 0.105 |  | 26.090 |  | 12 |  | 0.004 |  | 1.000 |  |
|  |  | VALIDATION Generative Models |  |  |  |  |  |  |  |  |  |  |  |
|  |  | TEST Regression Methods |  | -0.051 |  | 15.654 |  | 12 |  | -0.003 |  | 1.000 |  |
|  |  | TRAIN Regression Methods |  | 0.175 |  | 26.090 |  | 12 |  | 0.007 |  | 1.000 |  |
|  |  | VALIDATION Regression Methods |  |  |  |  |  |  |  |  |  |  |  |
|  |  | TEST SVM Model |  | -93.065 |  | 26.090 |  | 12 |  | -3.567 |  | 0.139 |  |
|  |  | TRAIN SVM Model |  | -75.365 |  | 26.090 |  | 12 |  | -2.889 |  | 0.490 |  |
|  |  | VALIDATION SVM Model |  |  |  |  |  |  |  |  |  |  |  |
| TRAIN Ensemble Methods |  | VALIDATION Ensemble Methods |  |  |  |  |  |  |  |  |  |  |  |
|  |  | TEST Generative Models |  |  |  |  |  |  |  |  |  |  |  |
|  |  | TRAIN Generative Models |  |  |  |  |  |  |  |  |  |  |  |
|  |  | VALIDATION Generative Models |  |  |  |  |  |  |  |  |  |  |  |
|  |  | TEST Regression Methods |  |  |  |  |  |  |  |  |  |  |  |
|  |  | TRAIN Regression Methods |  |  |  |  |  |  |  |  |  |  |  |
|  |  | VALIDATION Regression Methods |  |  |  |  |  |  |  |  |  |  |  |
|  |  | TEST SVM Model |  |  |  |  |  |  |  |  |  |  |  |
|  |  | TRAIN SVM Model |  |  |  |  |  |  |  |  |  |  |  |
|  |  | VALIDATION SVM Model |  |  |  |  |  |  |  |  |  |  |  |
| VALIDATION Ensemble Methods |  | TEST Generative Models |  |  |  |  |  |  |  |  |  |  |  |
|  |  | TRAIN Generative Models |  |  |  |  |  |  |  |  |  |  |  |
|  |  | VALIDATION Generative Models |  |  |  |  |  |  |  |  |  |  |  |
|  |  | TEST Regression Methods |  |  |  |  |  |  |  |  |  |  |  |
|  |  | TRAIN Regression Methods |  |  |  |  |  |  |  |  |  |  |  |
|  |  | VALIDATION Regression Methods |  |  |  |  |  |  |  |  |  |  |  |
|  |  | TEST SVM Model |  |  |  |  |  |  |  |  |  |  |  |
|  |  | TRAIN SVM Model |  |  |  |  |  |  |  |  |  |  |  |
|  |  | VALIDATION SVM Model |  |  |  |  |  |  |  |  |  |  |  |
| TEST Generative Models |  | TRAIN Generative Models |  |  |  |  |  |  |  |  |  |  |  |
|  |  | VALIDATION Generative Models |  |  |  |  |  |  |  |  |  |  |  |
|  |  | TEST Regression Methods |  |  |  |  |  |  |  |  |  |  |  |
|  |  | TRAIN Regression Methods |  |  |  |  |  |  |  |  |  |  |  |
|  |  | VALIDATION Regression Methods |  |  |  |  |  |  |  |  |  |  |  |
|  |  | TEST SVM Model |  |  |  |  |  |  |  |  |  |  |  |
|  |  | TRAIN SVM Model |  |  |  |  |  |  |  |  |  |  |  |
|  |  | VALIDATION SVM Model |  |  |  |  |  |  |  |  |  |  |  |
| TRAIN Generative Models |  | VALIDATION Generative Models |  |  |  |  |  |  |  |  |  |  |  |
|  |  | TEST Regression Methods |  | -0.156 |  | 25.562 |  | 12 |  | -0.006 |  | 1.000 |  |
|  |  | TRAIN Regression Methods |  | 0.070 |  | 33.001 |  | 12 |  | 0.002 |  | 1.000 |  |
|  |  | VALIDATION Regression Methods |  |  |  |  |  |  |  |  |  |  |  |
|  |  | TEST SVM Model |  | -93.170 |  | 33.001 |  | 12 |  | -2.823 |  | 0.553 |  |
|  |  | TRAIN SVM Model |  | -75.470 |  | 33.001 |  | 12 |  | -2.287 |  | 1.000 |  |
|  |  | VALIDATION SVM Model |  |  |  |  |  |  |  |  |  |  |  |
| VALIDATION Generative Models |  | TEST Regression Methods |  |  |  |  |  |  |  |  |  |  |  |
|  |  | TRAIN Regression Methods |  |  |  |  |  |  |  |  |  |  |  |
|  |  | VALIDATION Regression Methods |  |  |  |  |  |  |  |  |  |  |  |
|  |  | TEST SVM Model |  |  |  |  |  |  |  |  |  |  |  |
|  |  | TRAIN SVM Model |  |  |  |  |  |  |  |  |  |  |  |
|  |  | VALIDATION SVM Model |  |  |  |  |  |  |  |  |  |  |  |
| TEST Regression Methods |  | TRAIN Regression Methods |  | 0.226 |  | 25.562 |  | 12 |  | 0.009 |  | 1.000 |  |
|  |  | VALIDATION Regression Methods |  |  |  |  |  |  |  |  |  |  |  |
|  |  | TEST SVM Model |  | -93.014 |  | 25.562 |  | 12 |  | -3.639 |  | 0.122 |  |
|  |  | TRAIN SVM Model |  | -75.314 |  | 25.562 |  | 12 |  | -2.946 |  | 0.440 |  |
|  |  | VALIDATION SVM Model |  |  |  |  |  |  |  |  |  |  |  |
| TRAIN Regression Methods |  | VALIDATION Regression Methods |  |  |  |  |  |  |  |  |  |  |  |
|  |  | TEST SVM Model |  | -93.240 |  | 33.001 |  | 12 |  | -2.825 |  | 0.551 |  |
|  |  | TRAIN SVM Model |  | -75.540 |  | 33.001 |  | 12 |  | -2.289 |  | 1.000 |  |
|  |  | VALIDATION SVM Model |  |  |  |  |  |  |  |  |  |  |  |
| VALIDATION Regression Methods |  | TEST SVM Model |  |  |  |  |  |  |  |  |  |  |  |
|  |  | TRAIN SVM Model |  |  |  |  |  |  |  |  |  |  |  |
|  |  | VALIDATION SVM Model |  |  |  |  |  |  |  |  |  |  |  |
| TEST SVM Model |  | TRAIN SVM Model |  | 17.700 |  | 33.001 |  | 12 |  | 0.536 |  | 1.000 |  |
|  |  | VALIDATION SVM Model |  |  |  |  |  |  |  |  |  |  |  |
| TRAIN SVM Model |  | VALIDATION SVM Model |  |  |  |  |  |  |  |  |  |  |  |
|  | | | | | | | | | | | | | |
| *Note.*  P-value adjusted for comparing a family of 36 estimates. | | | | | | | | | | | | | |
| *Note.*  Some parameters were not estimable due to missingness. | | | | | | | | | | | | | |
